# Predicting Probability of Response to Tumor Necrosis Factor Inhibitors for Individual Patients With Ankylosing Spondylitis

**DOI:** 10.1001/jamanetworkopen.2022.2312

**Published:** 2022-03-15

**Authors:** Runsheng Wang, Abhijit Dasgupta, Michael M. Ward

**Affiliations:** 1 Division of Rheumatology, Columbia University Irving Medical Center, New York, New York; 2 Garden State Rheumatology Consultants, Union, New Jersey; 3 Intramural Research Program, National Institute of Arthritis and Musculoskeletal and Skin Diseases, National Institutes of Health, Bethesda, Maryland; 4 now with AstraZeneca, Rockville, Maryland

## Abstract

**Importance:**

Tumor necrosis factor inhibitors (TNFis) have revolutionized the management of ankylosing spondylitis (AS); however, the lack of notable clinical responses in approximately one-half of patients suggests important heterogeneity in treatment response. Identifying patients likely to respond or not respond to TNFis could provide opportunities to personalize treatment strategies.

**Objective:**

To develop models of the probability of short-term response to TNFi treatment in individual patients with active AS.

**Design, Setting, and Participants:**

This is a retrospective cohort study using data of the TNFi group (ie, treatment group) from 10 randomized clinical trials (RCTs) of TNFi treatment among patients with active AS, conducted from 2002 to 2016. Participants were adult patients with active AS who failed nonsteroidal anti-inflammatory drugs. Included RCTs were phase 3 and 4 studies that assessed the efficacy of an originator TNFi at week 12 and/or week 24, either compared with placebo or an antirheumatic drug. The cohort was divided into a training and a testing set. Data analysis was conducted from July 1, 2019, to November 30, 2020.

**Exposures:**

All included patients received an originator TNFi for at least 12 weeks.

**Main Outcomes and Measures:**

Outcomes included major response and no response based on the change of AS Disease Activity Score at 12 weeks. Machine learning algorithms were applied to estimate the probability of having major response and no response for individual patients.

**Results:**

The study included 1899 participants from 10 trials. The training set included 1207 individuals (mean [SD] age, 39 [12] years; 908 [75.2%] men), of whom 407 (33.7%) had major response and 414 (34.3%) had no response. In the reduced logistic regression models, accuracy was 0.74 for major response and 0.75 for no response. The probability of major response increased with higher C-reactive protein (CRP) level, patient global assessment (PGA), and Bath AS Disease Activity Index (BASDAI) question 2 score and decreased with higher body mass index (BMI) and Bath AS Functional Index (BASFI) score. The probability of no response increased with age and BASFI score, and decreased with higher CRP level, BASDAI question 2 score, and PGA. In the testing set (692 participants; mean [SD] age, 38 [11] years; 533 [77.0%] men), models demonstrated moderate to high accuracy.

**Conclusions and Relevance:**

In this cohort study, the probability of initial response to TNFi was predicted from baseline variables, which may facilitate personalized treatment decision-making.

## Key Points

**Question:**

Is it possible to predict short-term treatment response to tumor necrosis factor inhibitors (TNFis) accurately in patients with active ankylosing spondylitis (AS)?

**Findings:**

In a cohort study of individual participant data from 1899 patients in 10 randomized clinical trials of TNFis, models based on baseline clinical variables were developed and validated to predict the probability of having major response or having no response after 12 weeks of receiving TNFis, with moderate to high accuracy.

**Meaning:**

These findings suggest that the probability of initial response to TNFi treatment can be accurately predicted from baseline variables, which may facilitate personalized treatment decision-making.

## Teaser

This cohort study develops and validates models of the probability of short-term response to tumor necrosis factor inhibitor treatment in individual patients with active ankylosing spondylitis.

## Introduction

Ankylosing spondylitis (AS) also known as radiographic axial spondyloarthritis (axial SpA), is a chronic inflammatory condition characterized by inflammation in the spine, peripheral joints, and entheses and extra-articular manifestations such as uveitis, psoriasis, inflammatory bowel diseases, and aortitis.^1^ The treatment options for symptom control in patients with axial SpA have expanded dramatically in the past decades with the availability of tumor necrosis factor inhibitors (TNFis), interleukin-17 (IL-17) inhibitors, and Janus kinase (JAK) inhibitors, and TNFis are recommended when patients remain with active symptoms despite maximal tolerated nonsteroidal anti-inflammatory drug therapy.^2,3^ Although randomized clinical trials (RCTs) have shown that TNFis are efficacious in patients with AS, approximately one-half of patients do not achieve a notable improvement, suggesting important heterogeneity in treatment response.^4^ As clinical trials and most observational studies measure and report responses at the group level, limited guidance is available to predict treatment responses in individual patients.

In clinical practice, both patients and clinicians are interested in knowing how likely a patient is to achieve a significant response to TNFis so that treatment plans may be personalized. Several patient characteristics have been associated with a favorable response to TNFis in AS, including young age,^5,6,7,8,9^ short disease duration,^5,7,10^ male sex,^7,8,9,10^ human leukocyte antigen B27 (HLA-B27) positivity,^6,7,11^ and elevated inflammatory markers.^5,7,9,10,11,12^ Other features, such as body mass index (BMI; calculated as weight in kilograms divided by height in meters squared), have not been investigated, even though patients with obesity and other inflammatory arthritides have been found to have poorer responses to TNFis.^13,14,15^ More importantly, previous studies did not consider how combining these risk factors could affect the response to treatment. In addition, although previous studies have suggested factors associated with better response at the group level, none have estimated the likelihood of response using a probability score for an individual patient.

Identifying patients who are unlikely to respond to TNFis is as important as identifying responders. Such patients may be considered for only short trials of TNFi if the expectation of benefit is low or be considered for treatment with other classes of biologics, such as IL-17 inhibitors or JAK inhibitors.

The objective of this study was to develop and validate predictive models for short-term treatment response to TNFis in patients with active AS. Our goal was to provide a method to calculate probability scores of achieving major response or having no response after initiation of TNFi treatment for individual patients that was easy to apply and interpret in clinical practice.

## Methods

This is a retrospective cohort study using prospectively collected data from RCTs of TNFis in patients with active AS. We requested individual participant data from trial sponsors via 2 clinical data sharing platforms (Vivli and Yale University Open Data Access [YODA]) and aggregated the data in a pooled analysis. This study followed the Transparent Reporting of a Multivariable Prediction Model for Individual Prognosis or Diagnosis (TRIPOD) statement. The study was exempt from institutional review board approval and the requirement for informed consent, according to 45 CFR §46.

### Inclusion and Exclusion Criteria and Selection of RCTs

For individual patients, the main inclusion criteria were (1) adults with AS by the modified New York criteria; (2) individuals enrolled in a randomized, double-blind trial that assessed the efficacy of an originator TNFi at week 12 and/or week 24, either compared with placebo or an antirheumatic drug, such as sulfasalazine; (3) individuals randomly assigned to the TNFi group in the trial. Based on the inclusion criteria for participants, we included RCTs based on the following criteria: (1) RCTs registered at ClinicalTrials.gov by December 31, 2018, with a study status of complete and result status of has result; (2) RCTs evaluating the efficacy of a TNFi in adult patients with active AS; (3) AS defined as fulfilling modified New York criteria; and (4) RCTs using TNFis including originator adalimumab, certolizumab, etanercept, golimumab, or infliximab. Pooling data from different medications was justified because these medications are in the same class and target the same cytokine, have similar mechanisms of action, and are used interchangeably clinically. A prior network meta-analysis reported that these medications have similar short-term efficacy in treating AS.^16^

To ensure the homogeneity of the study population, we excluded studies of patients with non–radiographic axial spondyloarthritis (SpA) and other SpA. This exclusion was used to remove possible concerns that poor model performance might be attributable to the inclusion of patients with different predictors of response or likelihood of response. We excluded open-label studies and open-label extensions of blinded trials. Because data from the 2 platforms could not be downloaded for potential merging due to security provisions, we developed the models using data from 1 platform (Vivli; the training set) and performed external validation using data from the other platform (YODA; the testing set).

### Outcomes and Predictors

We examined 2 outcomes at week 12: (1) major response, defined as decrease in AS Disease Activity Score (ASDAS) of 2.0 or greater and (2) no response, defined as decrease in ASDAS of less than 1.1. ASDAS is a composite score measuring disease activity. It includes assessment of patient-reported total back pain, patient global assessment (PGA) of disease activity, peripheral joint pain and swelling, duration of morning stiffness, and C-reactive protein (CRP) level or erythrocyte sedimentation rate.^16^ Major improvement is defined as a decrease of 2.0 or greater, and clinically important improvement is defined as a decrease of 1.1 or greater.^17^ Major response in this study therefore equals achieving major improvement, with ASDAS decrease of 2.0 or greater at week 12, and no response as not achieving clinically important improvement at week 12. These definitions conform with current Assessment of Spondyloarthritis International Society–European Alliance of Associations for Rheumatology (ASAS-EULAR) recommendations for continuation of TNFi or switching to a different biologic after 12 weeks of TNFi.^3^ It is important to note that the converse of no response is achievement of ASDAS clinically important improvement. We used binary outcomes for the study because the treatment decision is binary (ie, continue or stop treatment).

We initially considered 27 clinical characteristics at trial baseline as potential predictors (eTable 1 in the Supplement), including demographic characteristics (age, sex); BMI; laboratory tests (HLA-B27 status, CRP level), disease characteristics (disease duration; history of uveitis, inflammatory bowel disease, and/or psoriasis; tender and swollen joint counts); concurrent use of sulfasalazine, methotrexate, or corticosteroids; and prior use of a TNFi. Patient-reported measures included individual Bath ankylosing spondylitis disease activity index (BASDAI) item scores, Bath ankylosing spondylitis functional index (BASFI) score, total back pain, nocturnal back pain, and PGA. We also considered the 36-Item Short Form Health Survey (SF-36) subscales of fatigue and mental health to tap the construct of fibromyalgia. We did not include comorbidities other than BMI or SpA-related conditions because there is no evidence that common comorbidities, such as hypertension and diabetes, affect the response to TNFis in patients with AS.

### Statistical Analysis

After aggregating participant data from all TNFi treatment groups by data platform, we categorized participants as achieving major response (yes or no), and no response (yes or no) at week 12 and used descriptive analysis to summarize the baseline characteristics of participants in each group. For the few patients who had missing outcome data at week 12, we used their value at the most recent prior evaluation (eg, week 8, week 6).

In the training set, HLA-B27 data was missing for 1.5% of participants, which we therefore converted to 2 complimentary variables, HLA-B27 positive (yes or no) and HLA-B27 negative (yes or no). Other variables had less than 1% missing values, and we used K-nearest neighbor to impute missing data. We excluded psoriasis, inflammatory bowel disease, and prior TNFi use as predictors because their prevalence in the training set was less than 5%.

Because 4 variables (tender joint count, swollen joint count, SF-36 fatigue subscale, and SF-36 mental health subscale) were reported in only some trials, we examined their variable importance in the subset of trials in which they were included, using both logistic regression (LR) and random forest (RF) models. In these preliminary analyses, none of these variables were among the top 10 predictors of major response, while only the tender joint count was among the top 10 predictors of no response (ranked eighth). We therefore omitted these as potential predictors, which allowed for testing of 21 predictors in all available trials. We optimized prediction models using 5 different machine learning algorithms, including LR, linear discriminant analysis, support vector machine, gradient boosting tree, and RF.

We used data on the Vivli platform as a training set for model development. For each algorithm, we used 5:1 cross-validation for tuning the hyperparameters based on average accuracy (eMethods in the Supplement). We then generated the model and examined the accuracy, receiver operating characteristic area under curve (ROC AUC), sensitivity, and specificity of the optimized model (eMethods in the Supplement). We followed the same procedure for each of the 5 machine learning algorithms. We considered AUCs greater than 0.714 to represent a large effect size.^18^ We considered values of sensitivity and specificity of at least 0.80 as high and those between 0.40 and 0.79 as moderate.^19^

Because collecting data on 21 predictors has associated information costs and may not always be feasible in clinical practice, we generated reduced models for clinic use. We used permutation feature importance to rank the importance of each variable.^20^ Permutation feature importance is defined as the decrease of model performance when the values of a given variable are randomly shuffled. Based on the importance scores, we selected the 5 most important variables for LR models and the 3 most important variables for RF models, and repeated the process of model development.

To assess how data from individual trials affected the models’ results, we recomputed the LR and RF models iteratively leaving out one of the 6 trials in the test set. We qualitatively compared model performance and variable importance rankings across these iterations.

We then externally evaluated the full and reduced models in testing set (YODA platform), and examined the accuracy, ROC AUC, sensitivity, and specificity of each model. We used bootstrapping to compute confidence intervals for the measurement statistics on 1000 random samples that included 75% of patients (with replacement) in the testing set.^21^ We also constructed calibration curves that compared the predicted probability of each response to the observed responses in 10 strata (or bins), ordered by the predicted probability. Data were extracted and analyzed using python version 3.5 and scikit-learn version 0.23.2.^22^

## Results

### Study Cohort

We included data on 1899 participants from 10 eligible RCTs of TNFis (eTable 2 in the Supplement) in patients with active AS in this analysis. These participant-level data were accessible via 2 data sharing platforms.^23,24,25,26,27,28,29,30,31,32^ Among these trials, 1207 participants who were assigned to the TNFi groups from 6 studies were eligible and included for model development (training set), and 692 participants from 4 studies were eligible and included for model validation (testing set).

Baseline characteristics of these participants are summarized in Table 1 and Table 2. In the training set (Table 1), the mean (SD) age was 39 (12) years, 908 (75.2%) were men, 1013 (83.9%) were HLA-B27 positive, and the median (IQR) disease duration was 5.1 (1.1-12.6) years. Mean (SD) BMI was 25.6 (5.0), and the median CRP level was 1.2 (0.4-2.6) mg/dL (to convert to milligrams per liter, multiply by 10). Major response was achieved by 407 participants (33.7%), and 414 (34.3%) had no response. Similarly, in the testing set (Table 2), the mean (SD) age was 38 (11) years, 533 (77.0%) were men, 600 (86.7%) were HLA-B27 positive, and the median (IQR) disease duration was 2.5 (0.7-7.6) years. The mean (SD) BMI was 25.6 (5.1), and the median (IQR) CRP level was 1.3 (0.5-2.8) mg/dL. Major response was achieved by 284 participants (40.1%), and 206 (29.7%) had no response.

**Table 1.** Baseline Characteristics of Eligible Participants in the Training Set

Characteristic	Patients, mean (SD)				
	All (n = 1207)	Major response		No response	
		Yes (n = 407 [33.7%])	No (n = 800 [66.3%])	Yes (n = 414 [34.3%])	No (n = 793 [65.7%])
Age, y	39 (11.7)	36.2 (11)	40.5 (11.8)	43.3 (12)	36.8 (11)
Male, No. (%)	908 (75.2)	399 (83.3)	569 (71.1)	273 (65.9)	635 (80.1)
Female, No. (%)	299 (24.8)	68 (16.7)	231 (28.9)	141 (34.1)	158 (19.9)
HLA B27 positivity, No. (%)	1013 (83.9)	365 (89.7)	648 (81.0)	309 (74.6)	704 (88.8)
Disease duration, median (IQR), y	5.1 (1.1-12.6)	4.5 (0.9-11.5)	5.3 (1.3-13.1)	5.7 (1.4-14.5)	4.9 (1.1-11.6)
BMI	25.6 (5.0)	24.7 (4.7)	26.1 (5.1)	26.7 (5.0)	25.1 (4.8)
CRP level, median (IQR), mg/dL	1.2 (0.4-2.6)	2.4 (1.3-4.1)	0.8 (0.4-1.7)	0.5 (0.4-1.3)	1.7 (0.8-3.1)
BASDAI score^a^					
Overall	6.0 (1.6)	6.4 (1.5)	5.8 (1.7)	5.8 (1.8)	6.1 (1.6)
Question 1	6.3 (2.0)	6.5 (2.0)	6.1 (2.1)	6.2 (2.1)	6.3 (2.0)
Question 2	7.0 (1.8)	7.5 (1.6)	6.7 (1.8)	6.6 (2.0)	7.2 (1.6)
Question 3	4.9 (2.8)	5.4 (2.7)	4.6 (2.8)	4.7 (2.8)	5.0 (2.8)
Question 4	5.5 (2.5)	6.1 (2.3)	5.2 (2.6)	5.3 (2.6)	5.7 (2.4)
Question 5	6.7 (2.1)	7.0 (2.0)	6.5 (2.1)	6.5 (2.2)	6.8 (2.0)
Question 6	6.0 (2.6)	6.2 (2.7)	5.9 (2.6)	5.9 (2.6)	6.0 (2.6)
BASFI score^a^	5.3 (2.2)	5.6 (2.1)	5.2 (2.2)	5.3 (2.2)	5.4 (2.2)
Night pain^a^	6.3 (2.2)	6.7 (2.2)	6.0 (2.2)	5.9 (2.3)	6.5 (2.2)
Total back pain^a^	6.4 (1.9)	6.8 (1.9)	6.3 (1.9)	6.2 (2.0)	6.6 (1.9)
Patient global assessment^a^	6.5 (1.9)	7.1 (1.7)	6.2 (1.9)	6.1 (2.0)	6.7 (1.8)
Spondyloarthritis-related conditions, No. (%)					
Uveitis	139 (11.5)	46 (11.3)	93 (11.6)	53 (12.8)	86 (10.8)
Psoriasis	56 (4.6)	20 (4.9)	35 (4.4)	19 (4.6)	36 (4.5)
Inflammatory bowel disease	23 (1.9)	10 (2.5)	13 (1.6)	10 (2.4)	13 (1.6)
Prior medication use, No. (%)					
Methotrexate use	202 (16.7)	74 (18.2)	128 (16.0)	72 (17.4)	130 (16.4)
Sulfasalazine use	304 (25.2)	103 (25.3)	201 (25.1)	108 (26.1)	196 (24.7)
Systemic corticosteroid	142 (11.8)	58 (14.3)	84 (10.5)	49 (11.8)	93 (11.7)
Prior TNFi exposure	0	0	0	0	0

Abbreviations: BASDAI, Bath ankylosing spondylitis disease activity index; BASFI, Bath ankylosing spondylitis function index; BMI, body mass index (calculated as weight in kilograms divided by height in meters squared); CRP, C-reactive protein; HLA, human leukocyte antigen; TNFi, tumor necrosis factor inhibitor.

SI conversion factor: To convert CRP to milligrams per liter, multiply by 10.

^a^ BASDAI score, BASFI score, night pain, total back pain, and patient global assessment range from 0 to 10.

**Table 2.** Baseline Characteristics of Eligible Participants in Testing Set

Characteristic	Patients, mean (SD)				
	All (n = 692)	Major response		No response	
		Yes (n = 284 [41.0%])	No (n = 437 [63.2%])	Yes (n = 206 [29.7%])	No (n = 408 [59.0%])
Age, y	37.7 (11.4)	35.1 (10.2)	38.9 (11.9)	41.2 (12.5)	39.6 (11.9)
Male, No. (%)	533 (77.0)	332 (76.0)	238 (83.8)	137 (66.5)	295 (72.3)
Female, No. (%)	159 (23.0)	104 (24.0)	49 (17.2)	69 (33.5)	113 (27.7)
HLA B27 positivity, No. (%)	600 (86.7)	368 (84.1)	258 (90.8)	162 (78.9)	341 (83.7)
Disease duration, median (IQR), y	2.5 (0.7-7.6)	2.1 (0.7-6.1)	2.5 (0.8-8)	3.3 (1-8.9)	2.6 (0.8-8)
BMI	25.6 (5.1)	24.5 (4.3)	25.8 (5.2)	26.8 (5.7)	26.3 (5.5)
CRP level, median (IQR), mg/dL	1.3 (0.5-2.8)	2.2 (1.2-4.1)	1.1 (0.5-2.5)	0.5 (0.2-1.1)	0.8 (0.3-1.6)
BASDAI score^a^					
Overall	6.7 (1.5)	6.9 (1.4)	6.7 (1.5)	6.5 (1.5)	6.6 (1.5)
Question 1	6.9 (1.8)	6.9 (1.9)	6.9 (1.8)	6.8 (1.9)	6.9 (1.8)
Question 2	7.6 (1.5)	7.8 (1.5)	7.5 (1.6)	7.4 (1.7)	7.5 (1.6)
Question 3	5.7 (2.7)	6.0 (2.6)	5.8 (2.7)	5.5 (2.8)	5.5 (2.7)
Question 4	6.3 (2.3)	6.6 (2.3)	6.2 (2.4)	6.0 (2.4)	6.2 (2.3)
Question 5	7.2 (1.9)	7.4 (1.9)	7.1 (2.0)	7.1 (2.0)	7.1 (1.9)
Question 6	6.5 (2.7)	6.7 (2.6)	6.5 (2.8)	6.4 (2.8)	6.4 (2.7)
BASFI score^a^	5.8 (2.1)	5.7 (2)	5.8 (2.2)	5.8 (2.2)	5.8 (2.1)
Night pain^a^	6.7 (2.1)	6.9 (2)	6.6 (2.2)	6.6 (2.2)	6.5 (2.2)
Total back pain^a^	6.7 (1.9)	6.9 (1.9)	6.7 (2)	6.7 (1.9)	6.6 (1.9)
Patient global assessment^a^	7.1 (1.6)	7.3 (1.5)	7 (1.7)	6.8 (1.7)	6.9 (1.7)
Spondyloarthritis related conditions, No. (%)					
Uveitis	179 (25.9)	107 (24.5)	91 (31.9)	45 (21.8)	89 (21.7)
Psoriasis	46 (6.7)	28 (6.4)	13 (4.6)	20 (9.5)	33 (8.1)
Inflammatory bowel disease	37 (5.3)	21 (4.7)	17 (5.9)	10 (5.0)	20 (4.9)
Prior medication use, No. (%)					
Methotrexate use	115 (16.6)	87 (19.9)	46 (16.2)	35 (17.0)	69 (16.9)
Sulfasalazine use	202 (29.2)	133 (30.4)	99 (34.9)	50 (24.3)	103 (25.2)
Systemic corticosteroid	106 (15.3)	70 (16.0)	48 (16.9)	25 (12.1)	58 (14.2)
Prior TNFi exposure	16 (2.3)	8 (1.8)	8 (2.8)	1 (0.5)	8 (2.0)

Abbreviations: BASDAI, Bath ankylosing spondylitis disease activity index; BASFI, Bath ankylosing spondylitis function index; BMI, body mass index (calculated as weight in kilograms divided by height in meters squared); CRP, C-reactive protein; HLA, human leukocyte antigen; TNFi, tumor necrosis factor inhibitor.

SI conversion factor: To convert CRP to milligrams per liter, multiply by 10.

^a^ BASDAI score, BASFI score, night pain, total back pain, and patient global assessment range from 0 to 10.

### Model Development

We optimized prediction models using each of the 5 different machine learning algorithms in the training set, based on accuracy for predicting responses (that is, the proportion of patients correctly classified as improved or not by the model) using all 21 predictors. The overall performance of all 5 algorithms to predict major response and no response were similar (eTable 3 in the Supplement). In predicting Major Response, the accuracy ranged from 0.72 (gradient boosting tree) to 0.74 (LR). ROC AUC ranged from 0.79 (gradient boosting tree) to 0.81 (LR), with high specificity and moderate sensitivity. In predicting no response, the accuracy ranged from 0.73 (gradient boosting tree) to 0.75 (LR). ROC AUC ranged from 0.76 (gradient boosting tree) to 0.79 (linear discriminant analysis), with high specificity and moderate sensitivity.

Based on the overall similar performance among the methods, we continued the analysis using models based on LR and RF methods, which are more familiar to clinicians. We also used both methods to generate simplified (reduced) models for predicting major response and no response, using permutation feature importance to select a subset of variables with the greatest predictive ability. The Figure shows the ranking of the 10 most important features for each full model. For major response, the 5 most important features in the LR model were CRP level, PGA, BMI, BASFI score, and BASDAI question 2 score (eg, severity of spine pain). The probability of achieving a major response increased with higher CRP level, PGA, and BASDAI question 2 score, and decreased with higher BMI and BASFI score. Consistent with the findings in LR model, the 3 most important features in the RF model were CRP, BASDAI question 2 score, and BMI.

**Figure.**
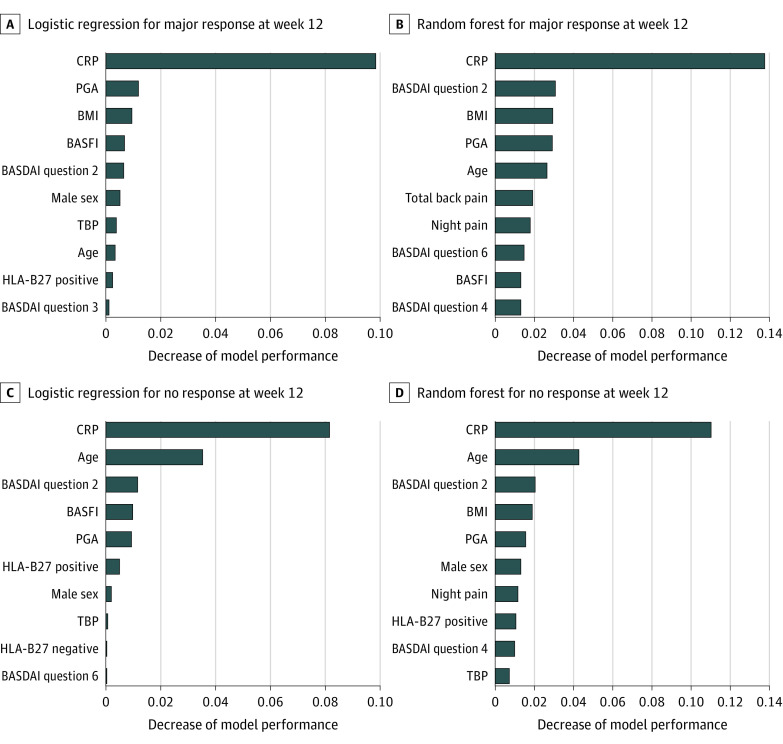
Permutation Feature Importance in Logistic Regression Models and Random Forest Models Each graph illustrates the ranking of the most important 10 variables in the corresponding model, and decrease of the model performance (ie, accuracy) when the variable is randomly shuffled. BASDAI indicates Bath ankylosing spondylitis disease activity index; BASFI, Bath ankylosing spondylitis function index; BMI, body mass index; CRP, C-reactive protein; HLA-B27, human leukocyte antigen B27; PGA, patient global assessment; TBP, total back pain.

For no response, the 5 most important features in the LR model were CRP level, age, BASDAI question 2 score, BASFI score, and PGA (Figure). The probability of having no response increased with older age and higher BASFI and decreased with higher CRP level, BASDAI question 2 score, and PGA. Again, consistent with the findings in LR model, the 3 most important features in the RF model were CRP level, age, and BASDAI question 2 score.

We generated reduced models using these smaller subsets of variables with 5:1 cross validation. The performance of the full models and reduced models was comparable (Table 3).

**Table 3.** Performance of Full (21-Variable) Models and Reduced (5- or 3-Variable) Models in the Training Set

Model	Accuracy	Sensitivity	Specificity	ROC AUC
**Major response at week 12**				
Logistic regression				
Full model	0.74	0.49	0.87	0.81
Reduced model	0.74	0.45	0.89	0.80
Random forest				
Full model	0.74	0.42	0.89	0.80
Reduced model	0.72	0.46	0.85	0.78
**No response at week 12**				
Logistic regression				
Full model	0.75	0.47	0.90	0.77
Reduced model	0.75	0.44	0.90	0.77
Random forest				
Full model	0.73	0.41	0.90	0.76
Reduced model	0.74	0.45	0.90	0.75

Abbreviation: ROC AUC, receiver operating characteristic area under curve.

In addition, we examined the performance of the full models after iteratively leaving one trial out of the training set (eTable 4 in the Supplement). Model performance was very similar across iterations (eTable 5 in the Supplement), suggesting that the results were not overly dependent on a single trial. The most important variables identified in these iterations were also quite similar to the analysis that included all trials (eFigures 1-4 and eTable 6 in the Supplement).

### Independent Validation

We then externally validated the full models and reduced models in the testing set (Table 4). Each model was tested in participants with complete data for all needed variables (ie, without imputation). Therefore, for the full models that use 21 variables for prediction, the sample size was 177, while for reduced models that use 3 or 5 variables for prediction, the sample sizes (range 625-692, depending on the model) were close to the entire testing set. The full models achieved moderate to high accuracy of 0.71 (RF model for major response) to 0.76 (RF model for no response), moderate ROC AUC of 0.65 (RF model for major response) to 0.68 (LR for no response), moderate sensitivity, and high specificity. Results for the reduced models were similar. The calibration curves demonstrated that the models predicted well at both low and high probabilities of major response and no response (eFigures 5 and 6 in the Supplement).

**Table 4.** External Validation of Full (21-Variable) Models and Reduced (5- or 3-Variable) Models

Model	Sample size, No.	Mean (95% CI)			
		Accuracy	Sensitivity	Specificity	ROC AUC
**Major response at week 12**					
Logistic regression					
Full model	177	0.71 (0.63-0.78)	0.54 (0.40-0.68)	0.82 (0.73-0.90)	0.67 (0.60-0.76)
Reduced model	625	0.70 (0.66-0.74)	0.49 (0.41-0.55)	0.85 (0.81-0.90)	0.67 (0.63-0.71)
Random forest					
Full model	177	0.69 (0.61-0.77)	0.47 (0.33-0.60)	0.83 (0.75-0.91)	0.65 (0.57-0.73)
Reduced model	691	0.70 (0.65-0.74)	0.47 (0.40-0.54)	0.85 (0.81-0.90)	0.66 (0.62-0.70)
**No response at week 12**					
Logistic regression					
Full model	177	0.76 (0.69-0.84)	0.41 (0.25-0.57)	0.92 (0.86-0.97)	0.66 (0.57-0.75)
Reduced model	625	0.74 (0.70-0.78)	0.33 (0.25-0.41)	0.92 (0.88-0.94)	0.62 (0.58-0.66)
Random forest					
Full model	177	0.76 (0.68-0.83)	0.36 (0.20-0.51)	0.93 (0.88-0.98)	0.65 (0.56-0.72)
Reduced model	692	0.77 (0.73-0.81)	0.38 (0.31-0.46)	0.93 (0.90-0.96)	0.66 (0.62-0.70

Abbreviation: ROC AUC, receiver operating characteristic area under curve.

At a major response prevalence of 0.25, the positive predictive values (PPV) for LR-based and RF-based models ranged from 0.49 to 0.60, and negative predictive values (NPV) ranged from 0.82 to 0.84 (eTable 7 in the Supplement). At a no response prevalence of 0.25, PPVs ranged from 0.61 to 0.77, and NPVs ranged from 0.81 to 0.83.

### Application

We created online calculators based on reduced LR models (only for review purpose; need further validation, not yet for use in clinical practice), which can be used to calculate the probability scores for major response or no response. For example, a patient with CRP level of 3.0 mg/dL, BMI of 20, PGA of 8 (of 10), BASDAI question 2 (back, neck, hip pain) score of 8 (of 10), and BASFI score of 3 (of 10) before starting TNFi, the probability of having major response after 12 weeks of TNFi treatment was predicted to be 73%, meaning the patient will most likely have a favorable response to TNFi. For another patient with CRP level of 1.0 mg/dL, age of 55 years, BASDAI question 2 score of 6 (of 10), PGA of 5 (of 10), and BASFI score of 7 (of 10) before starting TNFi, the probability of having no response is predicted to be 61%, meaning the patient will most likely have no response to TNFi after 12 weeks treatment.

## Discussion

Response to TNFi in patients with active AS is heterogenous, highlighting a need to better tailor treatment to patients based on their likelihood of response.^4^ We developed and validated predictive models that provide probability scores for major response and no response to TNFis after 12 weeks of treatment for individual patients with active AS.

Overall, our models demonstrated moderate to high accuracy and high specificity, using only information available at the start of treatment. Both the full and reduced models provided probability scores of having major response or no response at week 12, which could help clinicians and patients make personalized treatment decisions. The reduced models only included 3 or 5 variables that can be easily collected in clinical practice (CRP level, age, BMI, BASDAI question 2 score, BASFI score, and PGA), without specialized testing or documentation, which will facilitate clinical use. The 3-variable RF models do not require the PGA, which, in contrast to the BASDAI, is often not collected in routine clinical care of patients with AS.^33^

In the external testing cohort, the accuracy of different reduced models ranged from 0.70 to 0.78. Based on the sensitivity and specificity, at a prevalence of 25% for major response, the PPVs ranged from 0.49 to 0.60 and the NPVs from 0.82 to 0.84. These results indicated that, for a given patient, if the predicted probability of having major response was more than 50%, the patient may or may not have a major response to TNFis; whereas if the predicted probability was less than 50%, it is likely that the patient will not have a major response. Similarly, at a prevalence of 25% for no response, the PPVs ranged from 0.63 to 0.77 and the NPVs from 0.81 to 0.83. So, if the predicted probability of no response was more than 50%, the patient will possibly have no response, while if the predicted probability was less than 50%, the patient most likely will not have no response (ie, they would respond to treatment to some extent). Consequently, the PPVs are somewhat low when applied to a group of patients in which the prevalence of a true major response or no response is low, while the NPVs would be quite high in this scenario. However, it is important to note that these models and their applications need further investigation using practicing data in a clinical setting.

Consistent with previous findings,^5,7,9,10,11,12^ in our study, CRP level, BASDAI score, BASFI score, and age were among the most important predictors. In addition, we found higher BMI associated with lower probability of major response. Importantly, and in contrast to prior studies, our models integrated information from different risk factors to estimate the probability of treatment response for individual patients.

It is possible that prediction of the ASDAS-based responses may have been aided by the major contribution of the CRP level, which is heavily weighted in the ASDAS. It is important to note that even the reduced models for the ASDAS-based responses included variables not used in the calculation of the ASDAS, such as BMI, BASFI score, and age.

### Limitations

This study has some limitations. Smoking has been associated with poorer response to TNFis and shorter treatment adherence.^34^ Data on smoking were not available in some of the trials we analyzed. Second, all the participants in the training set were TNFi naive, and the models may not be similarly predictive in patients who had prior exposure to TNFis. Similarly, heterogeneity among patients included in the trials could have influenced the predictors and final models, although the iterative leave-out analysis suggests our results were robust to variations among trials. Third, we did not include nonsteroidal anti-inflammatory drug intake in our model, because data on their use were not consistent across studies. Fourth, our models have not been tested in daily clinical practice or in patients with a diagnosis of axial SpA. We focused on responses at 12 weeks because this is a common and recommended treatment decision point,^3^ but some patients may have delayed responses or low disease activity state. We did not include results of magnetic resonance imaging, because although these may enhance prediction, it would not be practical to obtain this imaging prior to starting TNFi treatment in all patients in clinical practice. Additionally, we do not know if pharmacogenomic data would enhance the prediction.

## Conclusions

The models developed and validated in this study provide probability scores for achieving major response or having no response to TNFi treatment among patients with AS; they can be used to facilitate personalized decision-making in clinical practice. Confidence in choosing TNFi treatment may be enhanced with a high probability score for major response. Absence of a response in a patient predicted to have a high probability may raise a question about adherence to treatment. Conversely, a course of TNFi treatment may be terminated quickly if nonresponse occurs in a patient predicted to have a high probability of no response. It will be important to develop similar models for response to other biologic treatments so treatment options can be prioritized based on a patient’s most likely response.

## Notes

Supplement. **eTable 1. **Variables Considered Potential Predictors
eMethods.
**eTable 2. **Randomized Clinical Trials of Tumor Necrosis Factors Inhibitors (TNFi) Included in the Current Study **eTable 3. **Performance of Different Machine Learning Models in Predicting Response to Tumor Necrosis Factor Inhibitors at Week 12 **eTable 4. **Characteristics of Participants Included in Each of 6 Subsets of Trials in the Testing Set, After Iteratively Omitting 1 Trial **eTable 5. **Performance of Logistic Regression and Random Forest Models in Predicting Major Response and No Response in the Testing Set, After Iteratively Omitting 1 of 6 Trials **eFigure 1.** Variable Importance Plots of Predictors of Major Response in 6 Iterations of Logistic Models in the Training Set, Each Based on a Different Subset of 5 Trials **eFigure 2.** Variable Importance Plots of Predictors of No Response in 6 Iterations of Logistic Models in the Training Set, Each Based on a Different Subset of 5 Trials **eFigure 3.** Variable Importance Plots of Predictors of Major Response in 6 Iterations of Random Forest Models in the Training Set, Each Based on a Different Subset of 5 Trials **eFigure 4.** Variable Importance Plots of Predictors of No Response in 6 Iterations of Random Forest Models in the Training Set, Each Based on a Different Subset of 5 Trials **eTable 6.** Consistency of Variable Importance Rankings in 6 Iterations of Models, Each Using a Different Subset of 5 Trials in the Training Set, With Variable Importance Rankings Based on Models Using All 6 Trials **eFigure 5.** Calibration Curves for Prediction of Major Response by the Logistic Regression and Random Forest Models **eFigure 6.** Calibration Curves for Prediction of No Response by the Logistic Regression and Random Forest Models **eTable 7.** Positive Predictive Values (PPVs) and Negative Predictive Values (NPVS) at Different Prevalences of Major Response and No Response
