# Supplementary material for: Predicting Probability of Response to Tumor Necrosis Factor Inhibitors for Individual Patients With Ankylosing Spondylitis
Source: JAMA Netw Open. 2022 Mar 15;5(3):e222312. doi: 10.1001/jamanetworkopen.2022.2312 (PMC8924712; doi:10.1001/jamanetworkopen.2022.2312)
Supplement: Supplement. — eTable 1. Variables Considered Potential Predictors eMethods. eTable 2. Randomized Clinical Trials of Tumor Necrosis Factors Inhibitors (TNFi) Included in the Current Study eTable 3. Performance of Different Machine Learning Models in Predicting Response to Tumor Necrosis Factor Inhibitors at Week 12 eTable 4. Characteristics of Participants Included in Each of 6 Subsets of Trials in the Testing Set, After Iteratively Omitting 1 Trial eTable 5. Performance of Logistic Regression and Random Forest Models in Predicting Major Response and No Response in the Testing Set, After Iteratively Omitting 1 of 6 Trials eFigure 1. Variable Importance Plots of Predictors of Major Response in 6 Iterations of Logistic Models in the Training Set, Each Based on a Different Subset of 5 Trials eFigure 2. Variable Importance Plots of Predictors of No Response in 6 Iterations of Logistic Models in the Training Set, Each Based on a Different Subset of 5 Trials eFigure 3. Variable Importance Plots of Predictors of Major Response in 6 Iterations of Random Forest Models in the Training Set, Each Based on a Different Subset of 5 Trials eFigure 4. Variable Importance Plots of Predictors of No Response in 6 Iterations of Random Forest Models in the Training Set, Each Based on a Different Subset of 5 Trials eTable 6. Consistency of Variable Importance Rankings in 6 Iterations of Models, Each Using a Different Subset of 5 Trials in the Training Set, With Variable Importance Rankings Based on Models Using All 6 Trials eFigure 5. Calibration Curves for Prediction of Major Response by the Logistic Regression and Random Forest Models eFigure 6. Calibration Curves for Prediction of No Response by the Logistic Regression and Random Forest Models eTable 7. Positive Predictive Values (PPVs) and Negative Predictive Values (NPVS) at Different Prevalences of Major Response and No Response [file jamanetwopen-e222312-s001.pdf]

Supplemental Online Content

Wang R, Dasgupta A, Ward MM. Predicting probability of response to tumor necrosis factor inhibitors for individual patients with ankylosing spondylitis. *JAMA Netw Open.* 2022;5(3):e222312. doi:10.1001/jamanetworkopen.2022.2312

eTable 1. Variables Considered Potential Predictors

eMethods.

eTable 2. Randomized Clinical Trials of Tumor Necrosis Factors Inhibitors (TNFi) Included in the Current Study

eTable 3. Performance of Different Machine Learning Models in Predicting Response to Tumor Necrosis Factor Inhibitors at Week 12

eTable 4. Characteristics of Participants Included in Each of 6 Subsets of Trials in the Testing Set, After Iteratively Omitting 1 Trial

eTable 5. Performance of Logistic Regression and Random Forest Models in Predicting Major Response and No Response in the Testing Set, After Iteratively Omitting 1 of 6 Trials

eFigure 1. Variable Importance Plots of Predictors of Major Response in 6 Iterations of Logistic Models in the Training Set, Each Based on a Different Subset of 5 Trials

eFigure 2. Variable Importance Plots of Predictors of No Response in 6 Iterations of Logistic Models in the Training Set, Each Based on a Different Subset of 5 Trials

eFigure 3. Variable Importance Plots of Predictors of Major Response in 6 Iterations of Random Forest Models in the Training Set, Each Based on a Different Subset of 5 Trials

eFigure 4. Variable Importance Plots of Predictors of No Response in 6 Iterations of Random Forest Models in the Training Set, Each Based on a Different Subset of 5 Trials

eTable 6. Consistency of Variable Importance Rankings in 6 Iterations of Models, Each Using a Different Subset of 5 Trials in the Training Set, With Variable Importance Rankings Based on Models Using All 6 Trials

eFigure 5. Calibration Curves for Prediction of Major Response by the Logistic Regression and Random Forest Models

eFigure 6. Calibration Curves for Prediction of No Response by the Logistic Regression and Random Forest Models

eTable 7. Positive Predictive Values (PPVs) and Negative Predictive Values (NPVS) at Different Prevalences of Major Response and No Response

This supplemental material has been provided by the authors to give readers additional information about their work.

© 2022 Wang R et al. *JAMA Network Open.*

eTable 1. Variables Considered Potential Predictors*

Variable	Status
Age	Included
Sex	Included
Body mass index	Included
HLA-B27 known positive	Included
HLA-B27 known negative	Included
Duration of ankylosing spondylitis	Included
Prior uveitis	Included
Prior inflammatory bowel disease	Excluded; prevalence < 5%
Prior psoriasis	Excluded; prevalence < 5%
Concurrent sulfasalazine use	Included
Concurrent methotrexate use	Included
Concurrent corticosteroid use	Included
BASDAI question 1 (fatigue)	Included
BASDAI question 2 (spinal pain)	Included
BASDAI question 3 (peripheral joint symptoms)	Included
BASDAI question 4 (joint tenderness)	Included
BASDAI question 5 (morning stiffness severity)	Included
BASDAI question 6 (morning stiffness duration)	Included
Bath ankylosing spondylitis functional index (BASFI)	Included
Patient global assessment	Included
Patient reported total back pain	Included
Patient reported nocturnal back pain	Included
Short-form 36 fatigue subscale	Excluded; not available in all trials
Short-form 36 mental health subscale	Excluded; not available in all trials
Tender joint count	Excluded; not available in all trials
Swollen joint count	Excluded; not available in all trials
C-reactive protein level	Included
Prior tumor necrosis factor inhibitor use	Excluded; prevalence < 5%

*BASDAI = Bath Ankylosing Spondylitis Disease Activity Index

© 2022 Wang R et al. *JAMA Network Open.*

eMethods.

1. Explanation of 5:1 cross validation.

This process involved:

- 1) randomly splitting the data in 5:1 ratio;
- 2) developing model parameters with a given set of hyperparameters in the larger portion of the data (5/6);
- 3) examining the model performance (accuracy) in the smaller portion of the data (1/6);
- 4) repeating the process 6 times;
- 5) calculating average accuracy;
- 6) choosing the set of hyperparameters with the best average accuracy.

2. Explanation of performance metrics.

For each outcome:

- **Accuracy** refers to the proportion of patients correctly classified by the model (i.e. (true positives + true negatives)/all), when the probability threshold for positivity is 0.5.
- **Receiver Operating Characteristic Area Under the Curve (ROC AUC)** measures how well the model separates those with the outcome of interest from those without the outcome at different thresholds.
- **Sensitivity** indicates the proportion of predicted positive cases out of all positive cases.
- **Specificity** indicates the proportion of predicted negative cases out of all negative cases.

© 2022 Wang R et al. *JAMA Network Open.*

For example, for a given patient, when predicting Major Response, if the predicted probability is ≥ 0.5, it is considered as a positive case; if the patient responded, it is considered a true positive case; and if the patient did not respond, it is considered a false positive case. Similar definitions are applied for negative cases, when the predicated probability is less than 0.5, as well as for predicting No Response.

© 2022 Wang R et al. *JAMA Network Open.*

eTable 2. Randomized Clinical Trials of Tumor Necrosis Factors Inhibitors (TNFi) Included in the Current Study*

NCT number [Reference]	TNFi and dose	Comparator	Number of patients in TNFi arm (N = 1899)	Mean age, years	Proportion male	Mean duration of AS, years	Mean BASDAI at baseline
NCT00085644 [23]	Adalimumab 40 mg qo week	Placebo	208	41.7	75.5	11.3	6.3
NCT00195819 [24]	Adalimumab 40 mg qo week	Placebo	41	41.9	76.3	14.5	6.5
NCT01114880 [25]	Adalimumab 40 mg qo week	Placebo	229	30.1	80.8	8.1	6.0
NCT00247962 [26]	Etanercept 50 mg q week	Sulfasalazine	379	40.7	73.6	7.5	5.9
NCT00418548 [27]	Etanercept 50 mg q week or 25 mg twice weekly	Placebo	305	40.6	72.7	9.5	6.0
NCT00421915 [28]	Etanercept 25 mg twice weekly	Placebo	45	45.3	80.0	15.0	6.1
NCT00265083 [29]	Golimumab 50 mg or 100 mg every 4 weeks	Placebo	278	38.0	71.9	5.2	6.8
NCT01248793 [30]	Golimumab 50 mg every 4 weeks	Placebo	108	30.5	83.3	4.2	6.6
NCT02186873 [31]	Golimumab 2 mg/kg intravenously x 3 over 12 weeks	Placebo	105	38.4	81.9	5.6	7.0
NCT00207701 [32]	Infliximab 5mg/kg intravenously x 3 over 12 weeks	Placebo	201	40.0	78.1	7.7	6.6

*NCT = National Clinical Trial; AS = ankylosing spondylitis; BASDAI = Bath AS Disease Activity Index; qo = every other.

© 2022 Wang R et al. *JAMA Network Open.*

eTable 3. Performance of Different Machine Learning Models in Predicting Response to Tumor Necrosis Factor Inhibitors at Week 12

	Accuracy	Sensitivity	Specificity	ROC AUC*
**Major Response**				
Logistic Regression	0.74	0.49	0.87	0.81
Supporting Vector Machine	0.74	0.53	0.85	0.81
Linear Discriminant Analysis	0.74	0.52	0.86	0.81
Random Forest	0.74	0.44	0.88	0.79
Gradient Boosting	0.72	0.46	0.86	0.79
**No Response**				
Logistic Regression	0.75	0.47	0.90	0.77
Support Vector Machine	0.73	0.48	0.86	0.77
Linear Discriminant Analysis	0.74	0.51	0.86	0.79
Random Forest	0.74	0.43	0.90	0.76
Gradient Boosting	0.73	0.44	0.87	0.76

*ROC AUC: receiver operating characteristic area under curve.

© 2022 Wang R et al. *JAMA Network Open.*

eTable 4. Characteristics of Participants Included in Each of 6 Subsets of Trials in the Testing Set, After Iteratively Omitting 1 Trial

Values are mean (standard deviation) unless otherwise noted.

Iteration	Trial omitted	Number of subjects	Age, years	Body mass index	Patient global assessment	BASDAI question 2*	BASFI*	Major response (%)	No response (%)
1	NCT00421915	1162	38.8 (11.7)	25.6 (5.0)	6.5 (1.9)	6.9 (1.8)	5.3 (2.2)	34	34
2	NCT00418548	899	38.5 (12.0)	25.5 (5.1)	6.4 (1.9)	7.0 (1.8)	5.1 (2.2)	34	34
3	NCT00247962	828	38.3 (11.6)	25.2 (5.0)	6.5 (1.9)	7.0 (1.7)	5.2 (2.2)	36	33
4	NCT00195819	1169	39.0 (11.7)	25.6 (5.0)	6.5 (1.9)	7.0 (1.7)	5.3 (2.2)	34	34
5	NCT00085644	999	38.5 (11.6)	25.3 (4.8)	6.5 (1.9)	6.9 (1.7)	5.4 (2.1)	34	33
6	NCT01114880	978	41.1 (11.3)	26.5 (4.8)	6.5 (1.9)	7.0 (1.8)	5.6 (2.1)	31	37

*BASDAI = Bath Ankylosing Spondylitis Disease Activity Index; BASFI = Bath Ankylosing Spondylitis Functional Index

© 2022 Wang R et al. *JAMA Network Open.*

eTable 5. Performance of Logistic Regression and Random Forest Models in Predicting Major Response and No Response in the Testing Set, After Iteratively Omitting 1 of 6 Trials

Logistic regression

	Iteration	Major response				No response			
		Accuracy	Sensitivity	Specificity	ROC AUC	Accuracy	Sensitivity	Specificity	ROC AUC
	1	0.74	0.45	0.88	0.80	0.76	0.50	0.80	0.79
	2	0.72	0.49	0.84	0.80	0.74	0.44	0.90	0.75
	3	0.73	0.55	0.83	0.80	0.76	0.52	0.87	0.79
	4	0.74	0.52	0.85	0.81	0.75	0.46	0.90	0.77
	5	0.74	0.50	0.87	0.82	0.76	0.45	0.91	0.77
	6	0.76	0.51	0.87	0.83	0.73	0.49	0.88	0.77

Random forest

	Iteration	Major response				No response			
		Accuracy	Sensitivity	Specificity	ROC AUC	Accuracy	Sensitivity	Specificity	ROC AUC
	1	0.72	0.44	0.88	0.78	0.75	0.41	0.91	0.77
	2	0.68	0.38	0.85	0.75	0.71	0.39	0.87	0.74
	3	0.71	0.47	0.85	0.77	0.74	0.43	0.90	0.77
	4	0.72	0.43	0.87	0.80	0.74	0.37	0.92	0.78
	5	0.73	0.45	0.87	0.80	0.74	0.40	0.91	0.77
	6	0.74	0.40	0.90	0.81	0.71	0.46	0.87	0.76

© 2022 Wang R et al. *JAMA Network Open.*

eFigure 1. Variable Importance Plots of Predictors of Major Response in 6 Iterations of Logistic Models in the Training Set, Each Based on a Different Subset of 5 Trials

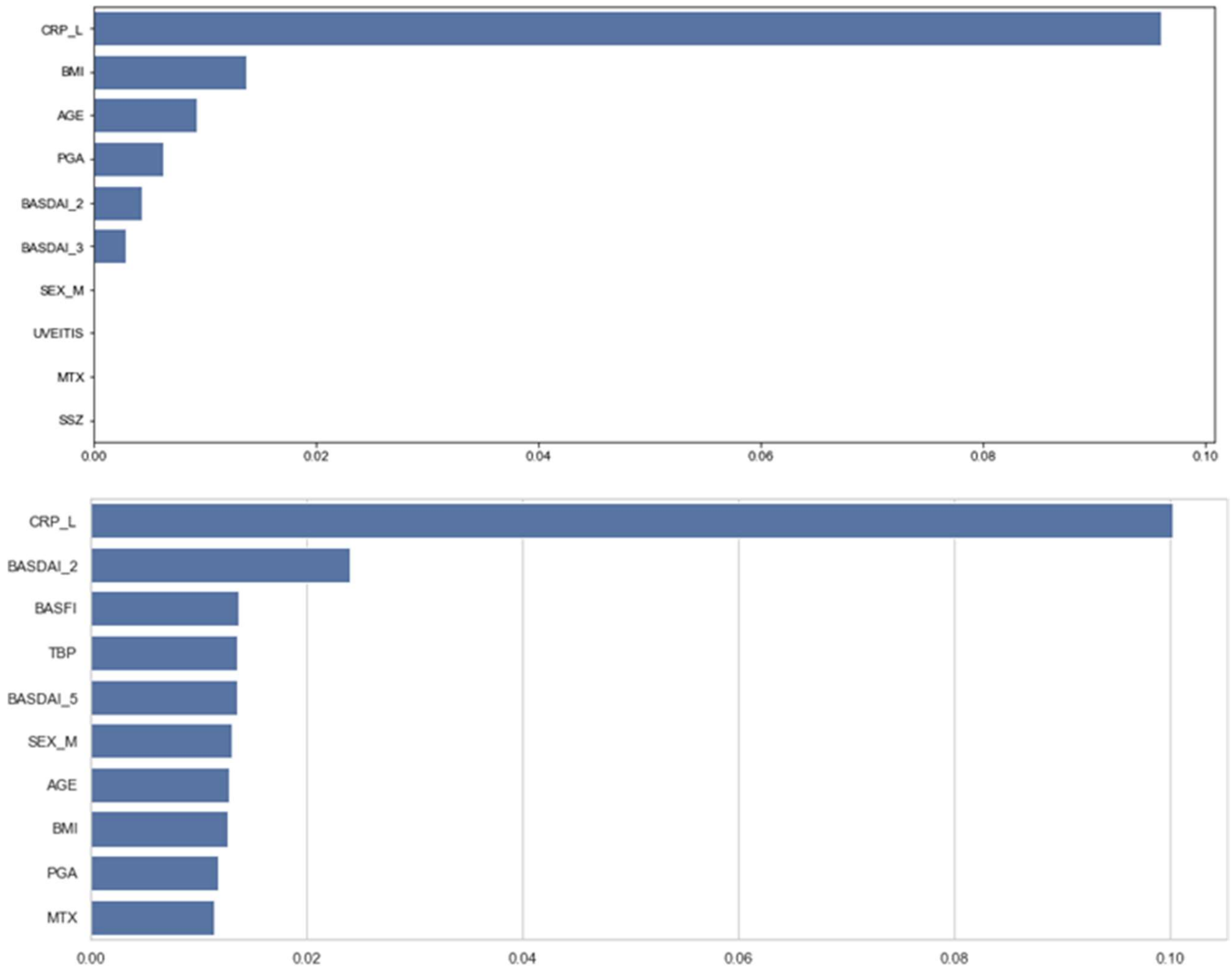

© 2022 Wang R et al. *JAMA Network Open.*

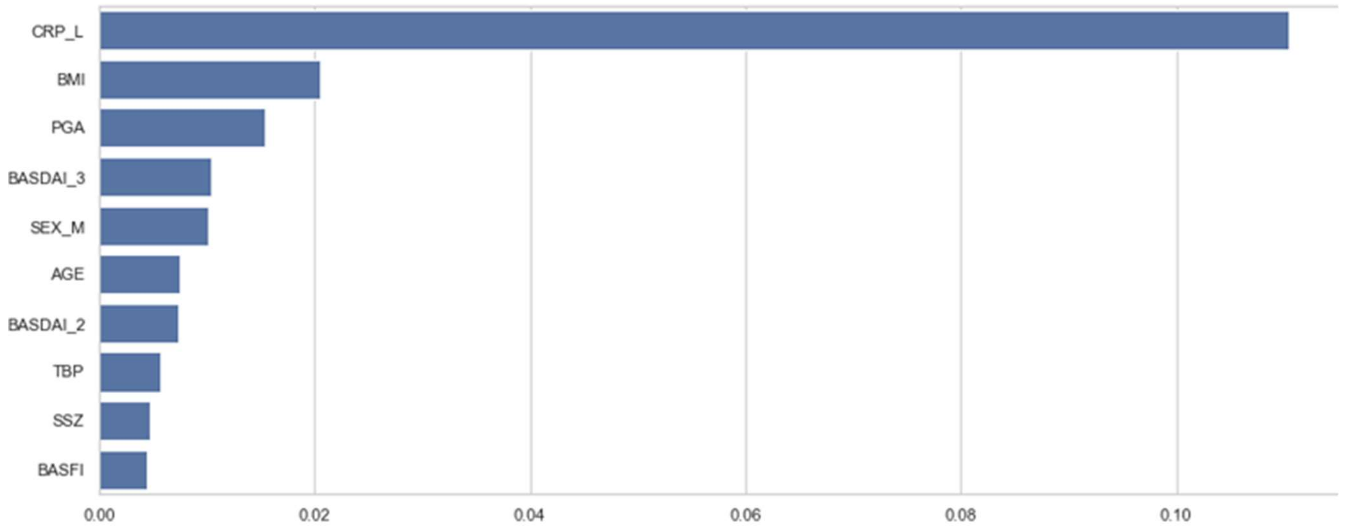

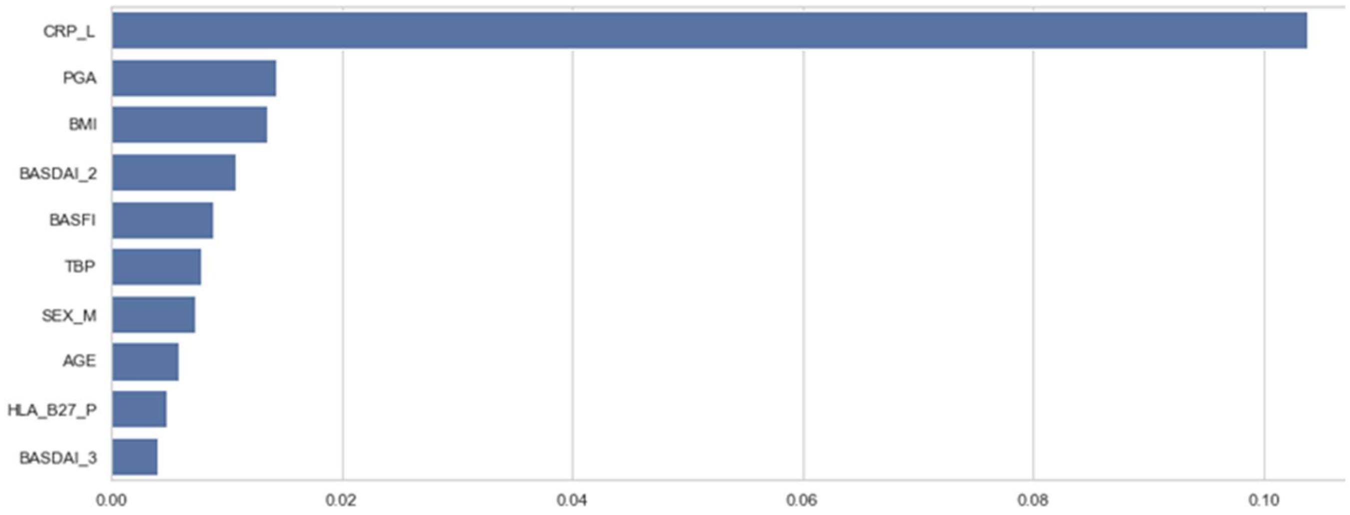

© 2022 Wang R et al. *JAMA Network Open.*

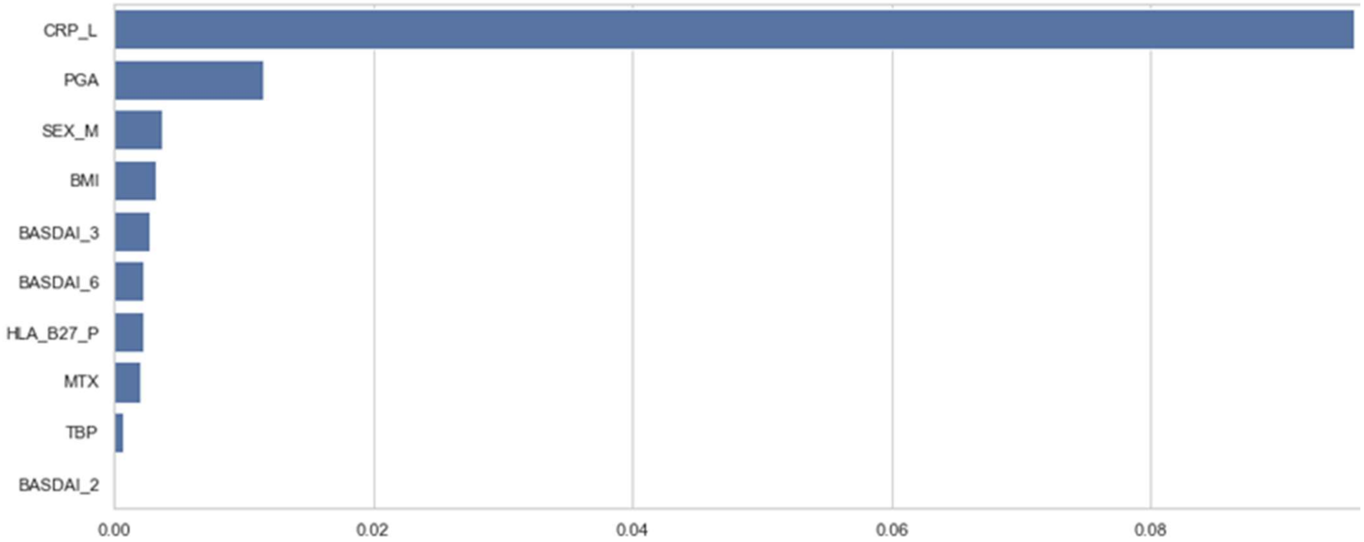

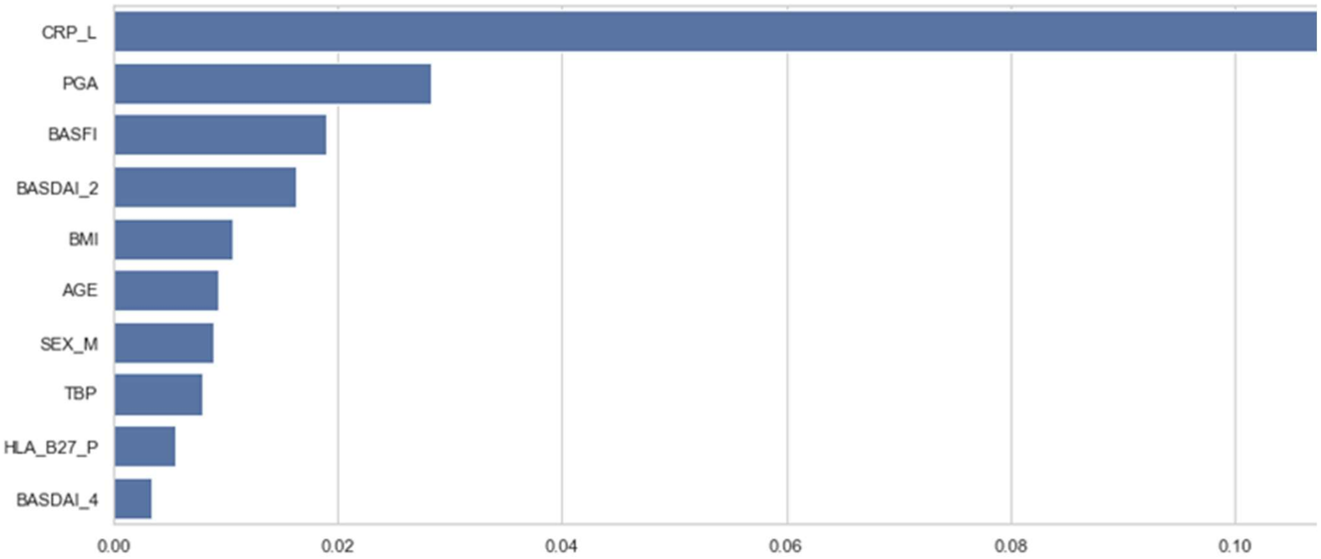

© 2022 Wang R et al. *JAMA Network Open.*

eFigure 2. Variable Importance Plots of Predictors of No Response in 6 Iterations of Logistic Models in the Training Set, Each Based on a Different Subset of 5 Trials

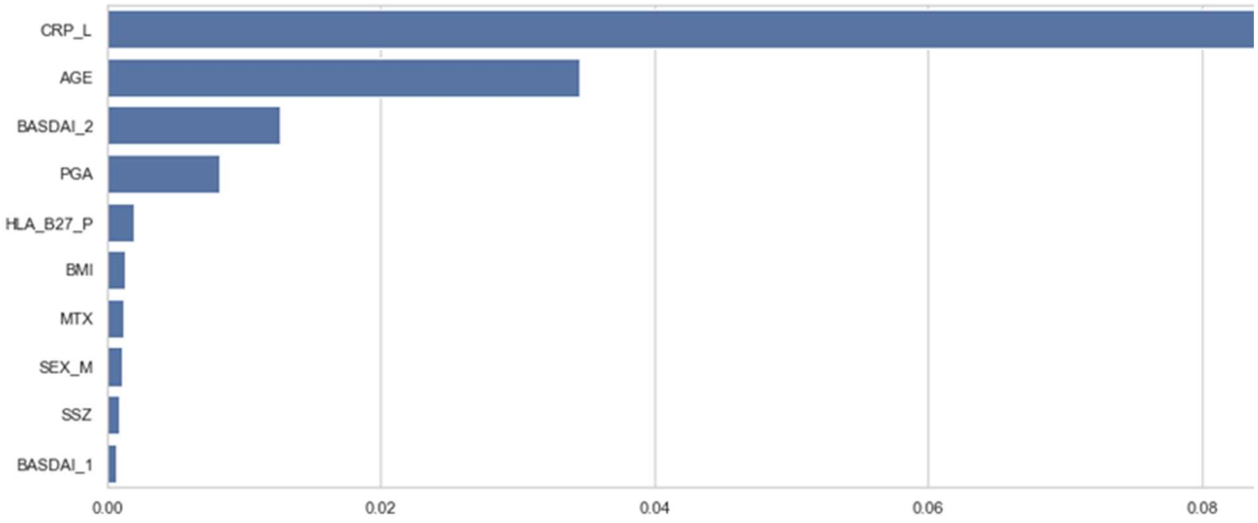

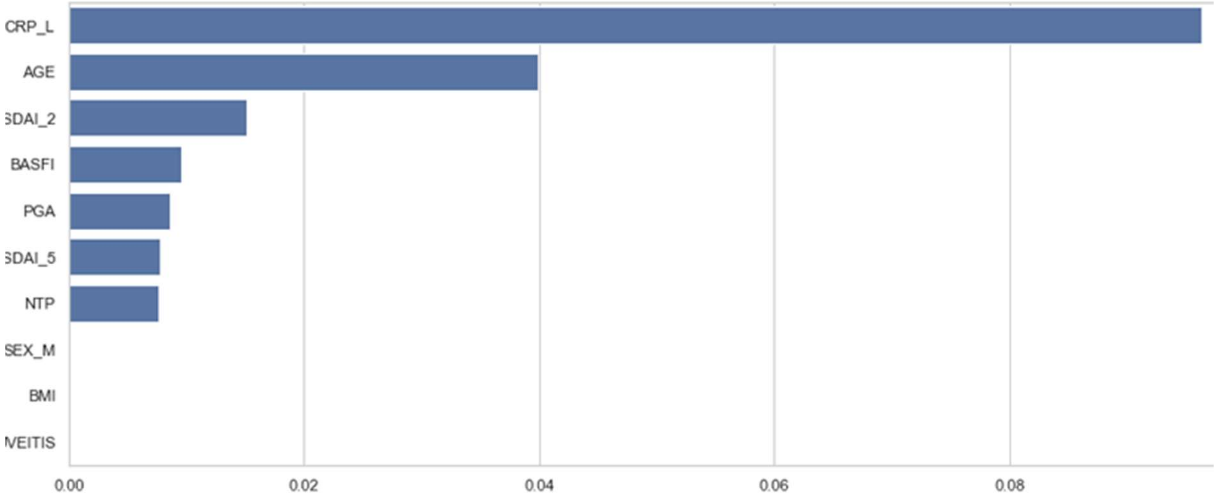

© 2022 Wang R et al. *JAMA Network Open.*

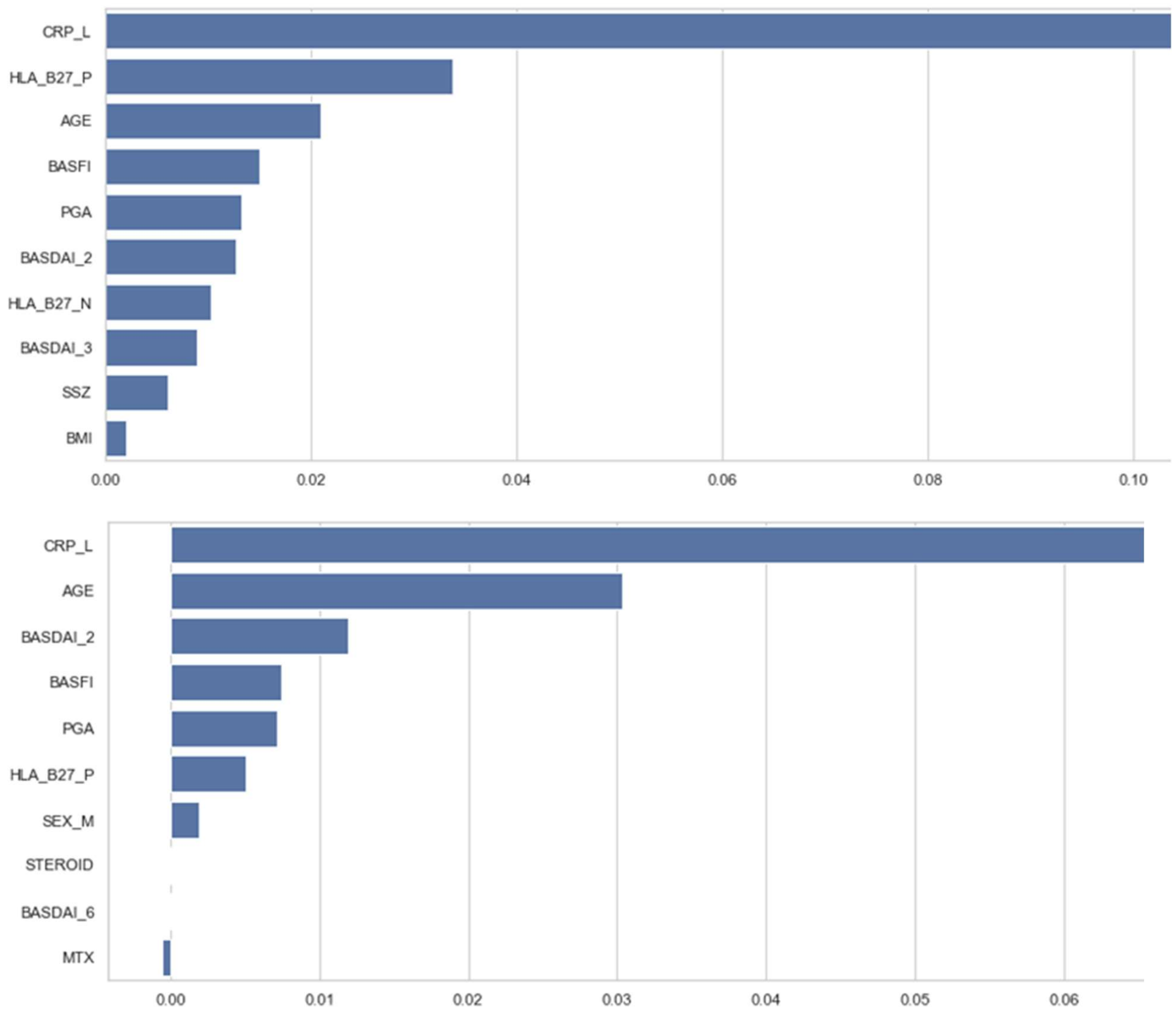

© 2022 Wang R et al. *JAMA Network Open.*

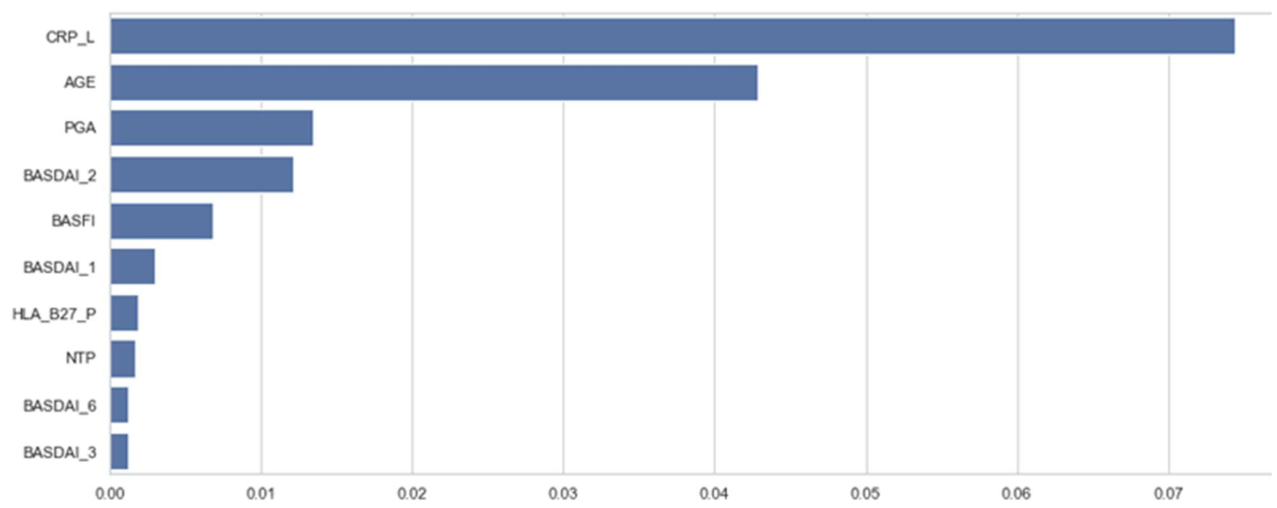

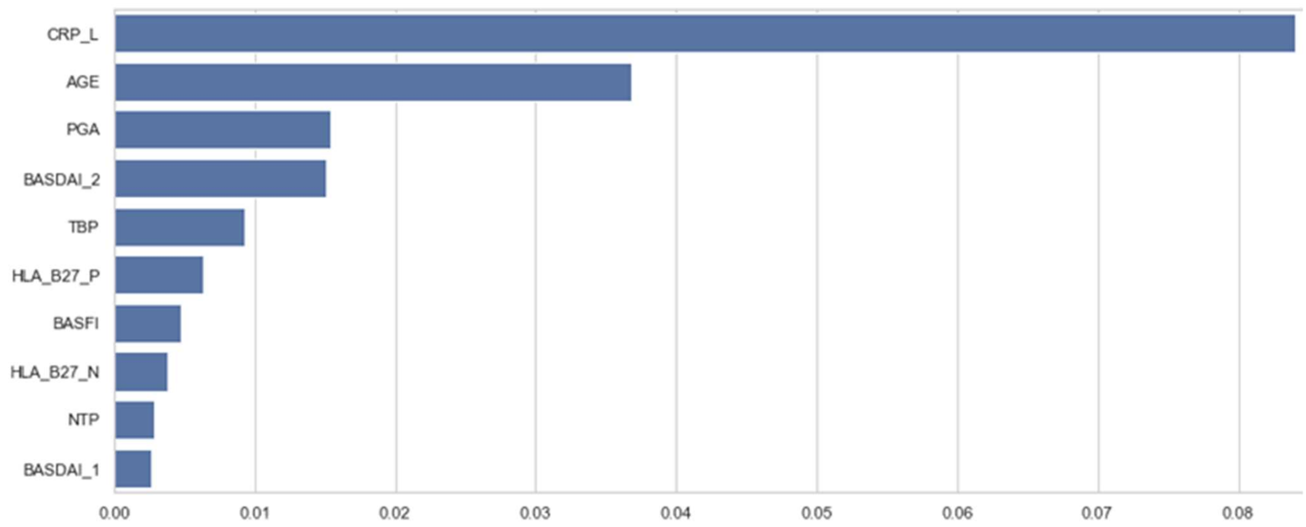

© 2022 Wang R et al. *JAMA Network Open.*

eFigure 3. Variable Importance Plots of Predictors of Major Response in 6 Iterations of Random Forest Models in the Training Set, Each Based on a Different Subset of 5 Trials

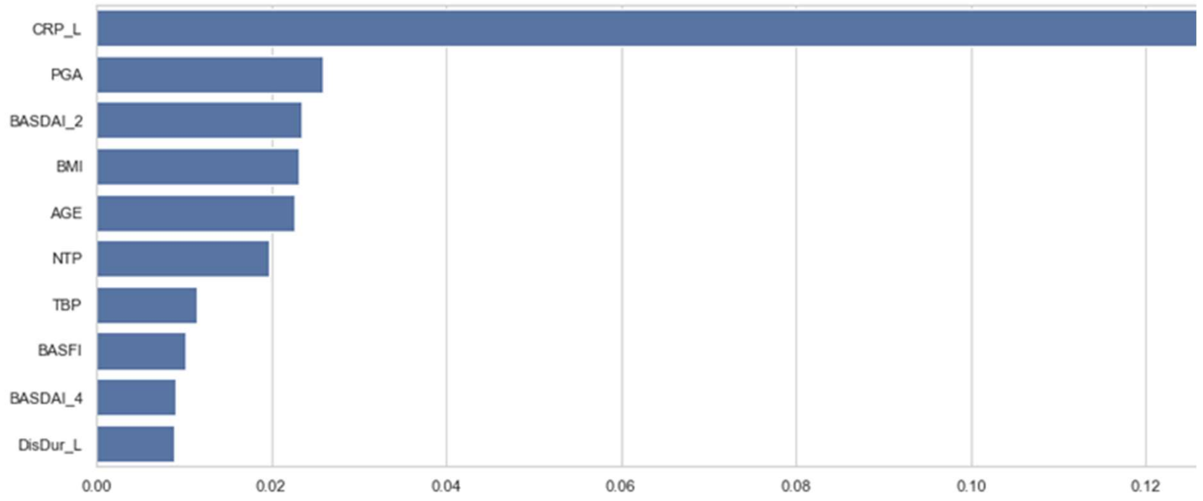

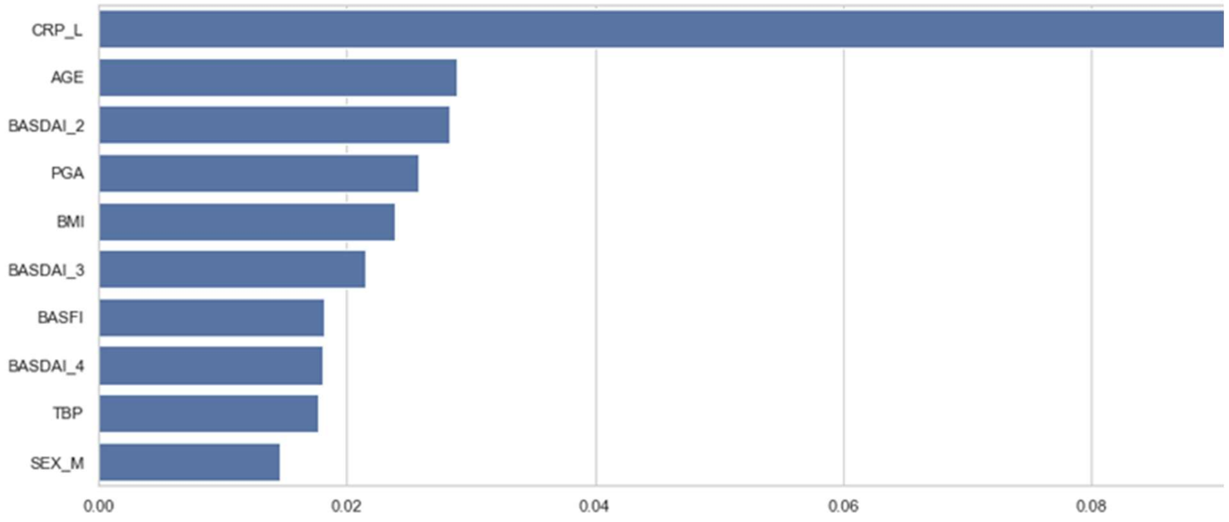

© 2022 Wang R et al. *JAMA Network Open.*

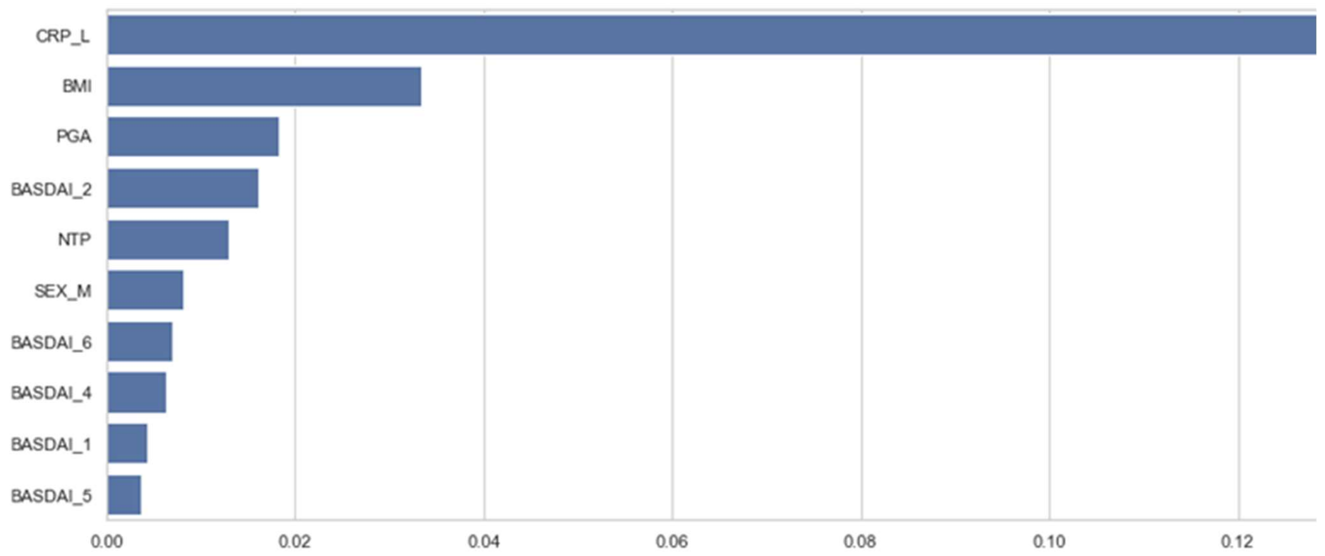

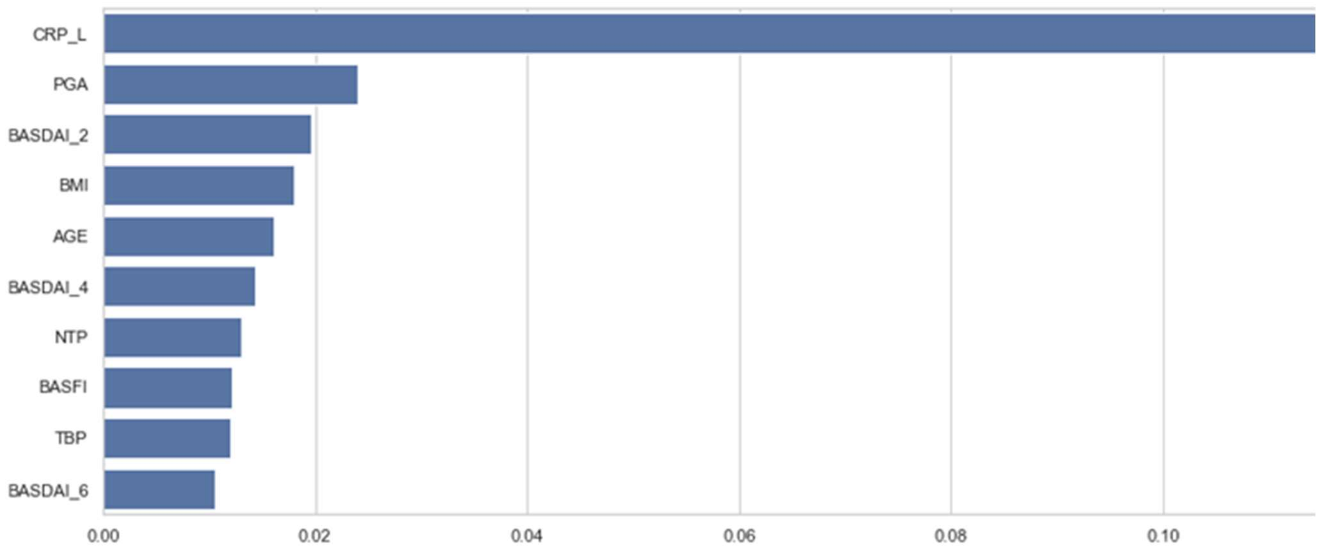

© 2022 Wang R et al. *JAMA Network Open.*

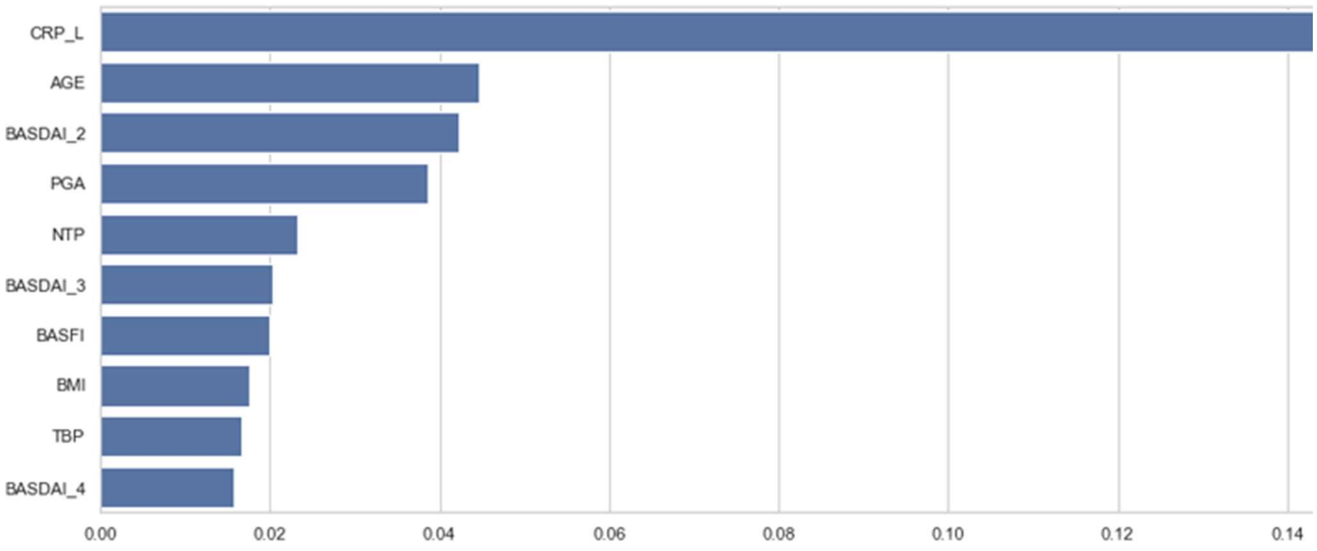

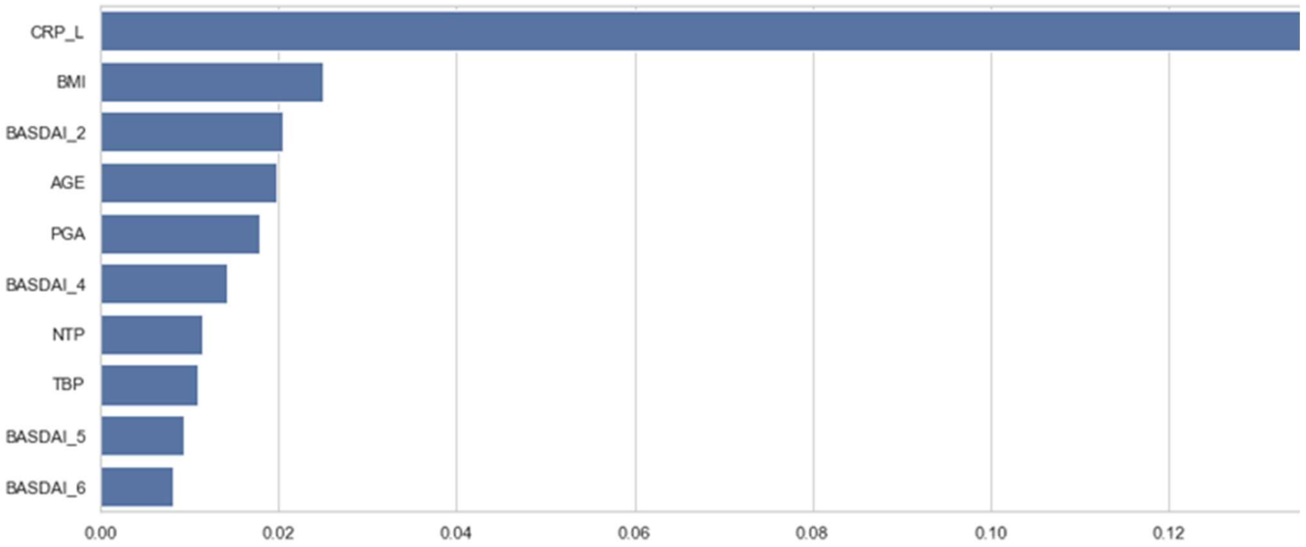

© 2022 Wang R et al. *JAMA Network Open.*

eFigure 4. Variable Importance Plots of Predictors of No Response in 6 Iterations of Random Forest Models in the Training Set, Each Based on a Different Subset of 5 Trials

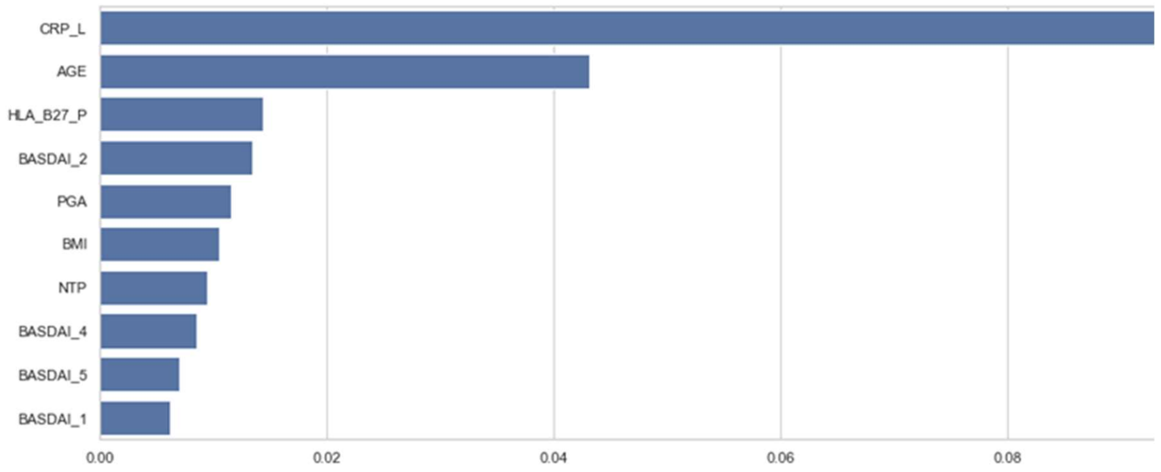

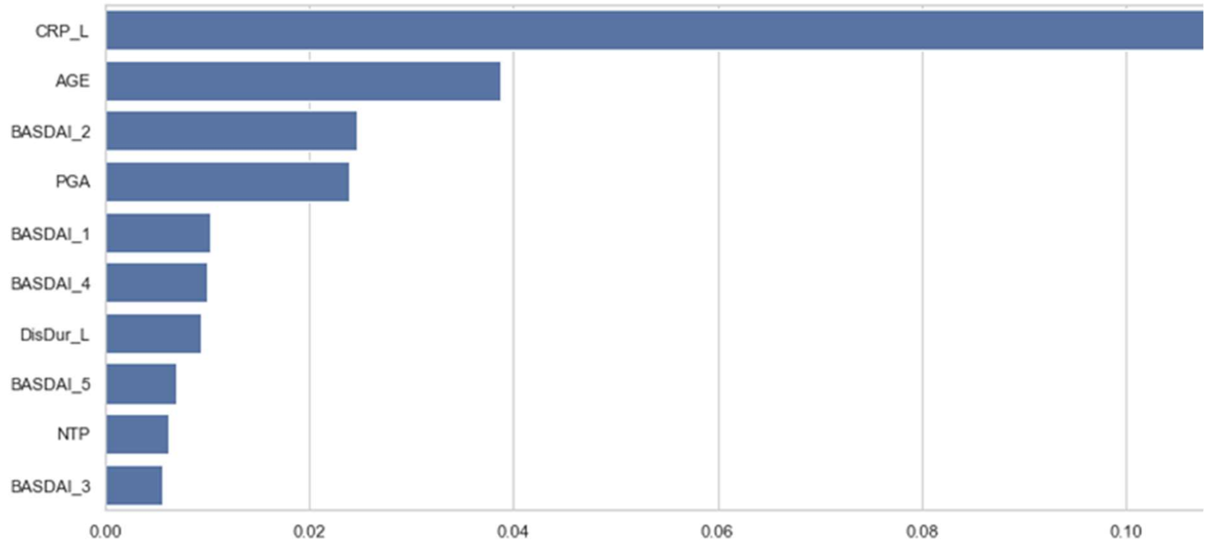

© 2022 Wang R et al. *JAMA Network Open.*

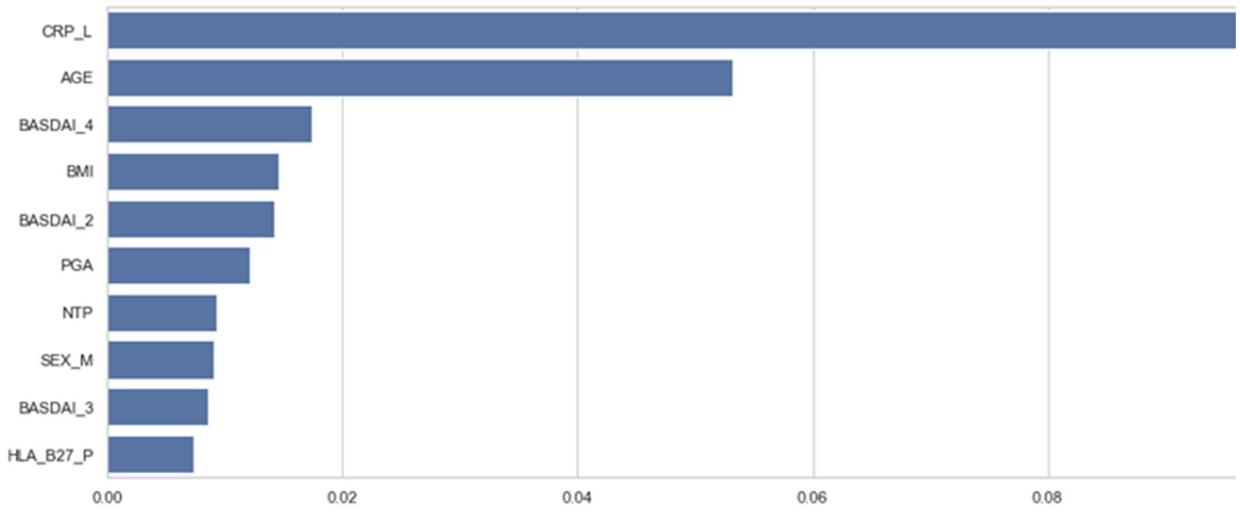

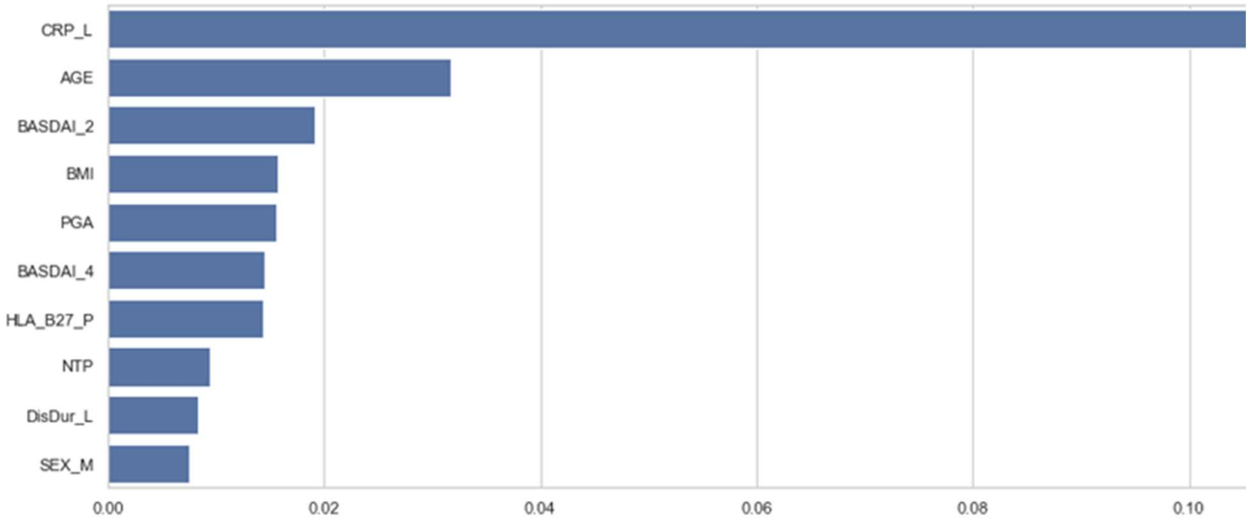

© 2022 Wang R et al. *JAMA Network Open.*

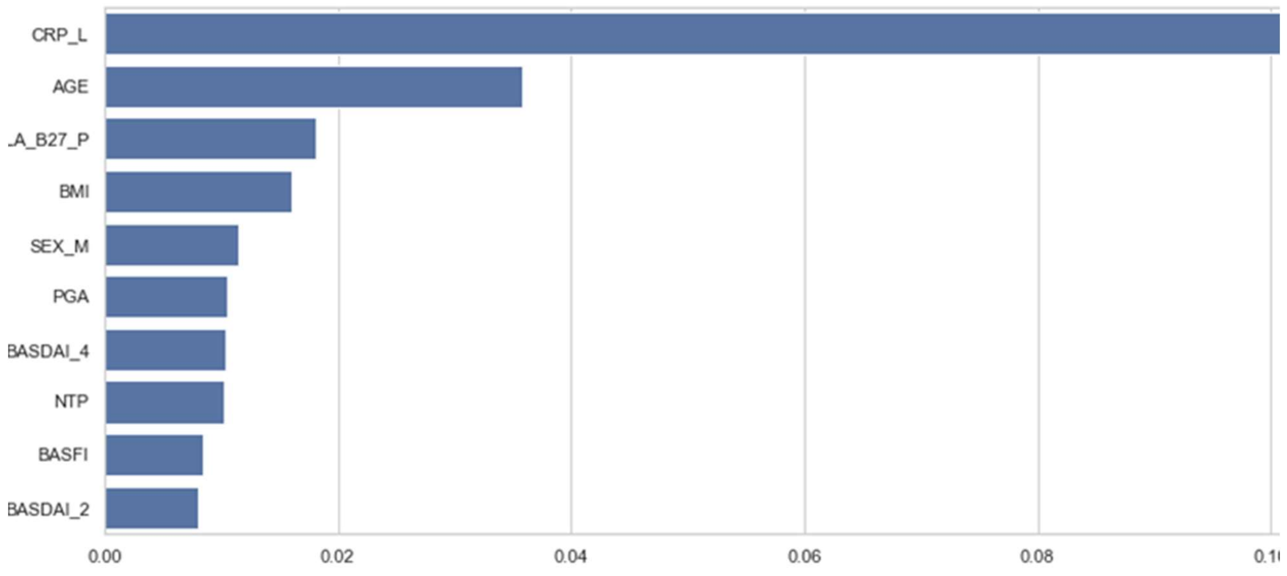

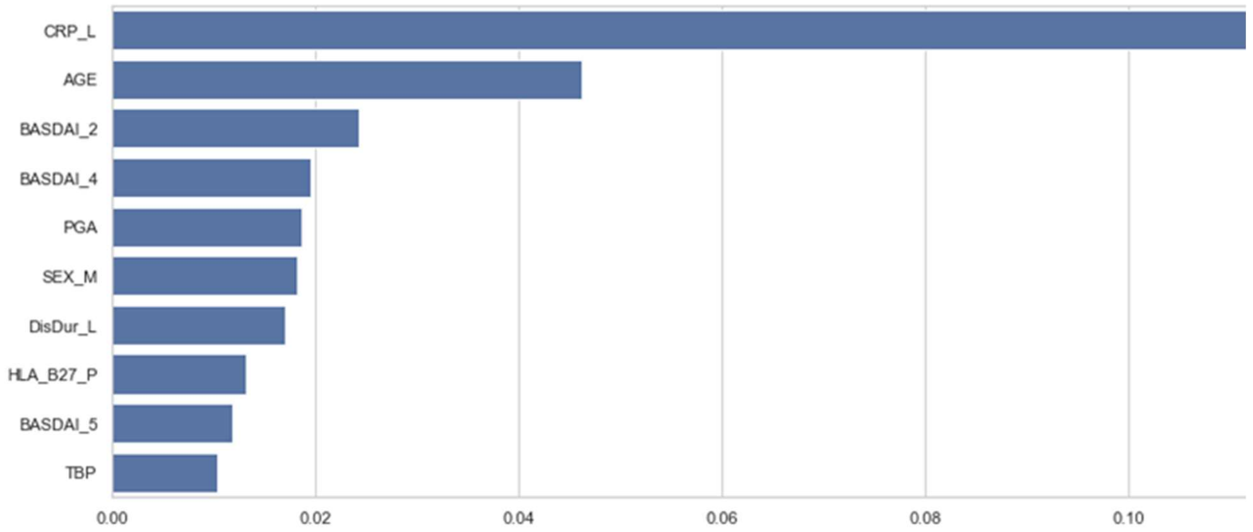

© 2022 Wang R et al. *JAMA Network Open.*

eTable 6. Consistency of Variable Importance Rankings in 6 Iterations of Models, Each Using a Different Subset of 5 Trials in the Training Set, With Variable Importance Rankings Based on Models Using All 6 Trials*

Model	Outcome	Most important variables in overall models	Number of iterations in which variable was among the five (or three) most important variables
Logistic regression	Major response	C-reactive protein	6/6
		Patient global assessment	5/6
		Body mass index	6/6
		BASFI	3/6
		BASDAI Q2	4/6
	No response	C-reactive protein	6/6
		Age	6/6
		BASDAI Q2	5/6
		BASFI	4/6
		Patient global assessment	6/6
Random forest	Major response	C-reactive protein	6/6
		Body mass index	4/6
		BASDAI Q2	5/6
	No response	C-reactive protein	6/6
		Age	6/6
		BASDAI Q2	4/6

*BASDAI = Bath Ankylosing Spondylitis Disease Activity Index; BASFI = Bath Ankylosing Spondylitis Functional Index

© 2022 Wang R et al. *JAMA Network Open.*

eFigure 5. Calibration Curves for Prediction of Major Response by the Logistic Regression and Random Forest Models

Each curve plots the predicted probability of Major Response versus the observed proportion of responders in ten strata, ordered by the probability of response. In models with good calibration, the curve should approximate the 45-degree line.

Logistic regression – Full Model

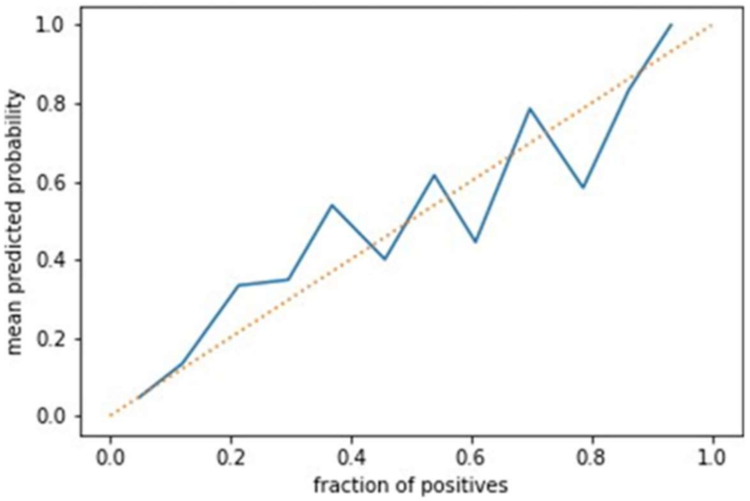

Logistic regression – Reduced Model

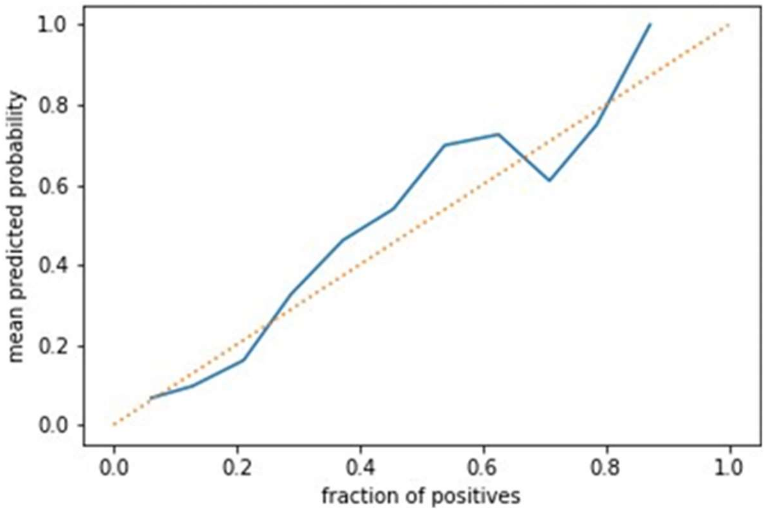

© 2022 Wang R et al. *JAMA Network Open.*

Random Forests – Full Model

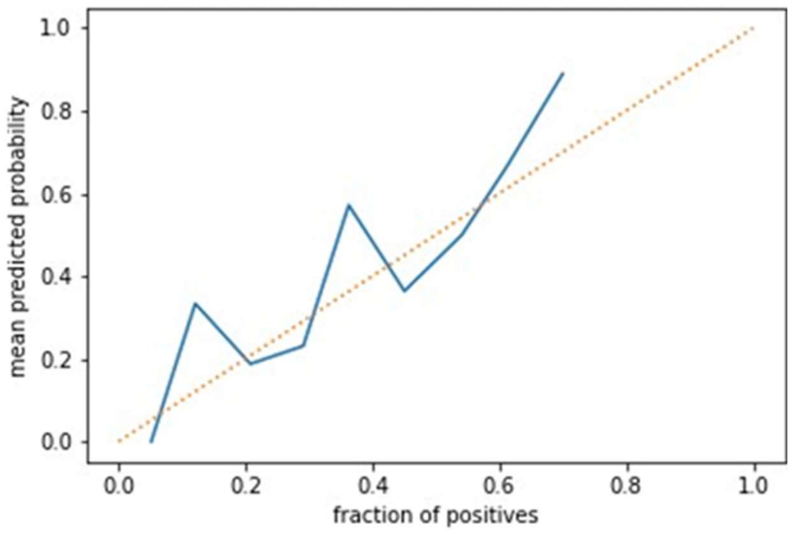

Random Forests – Reduced Model

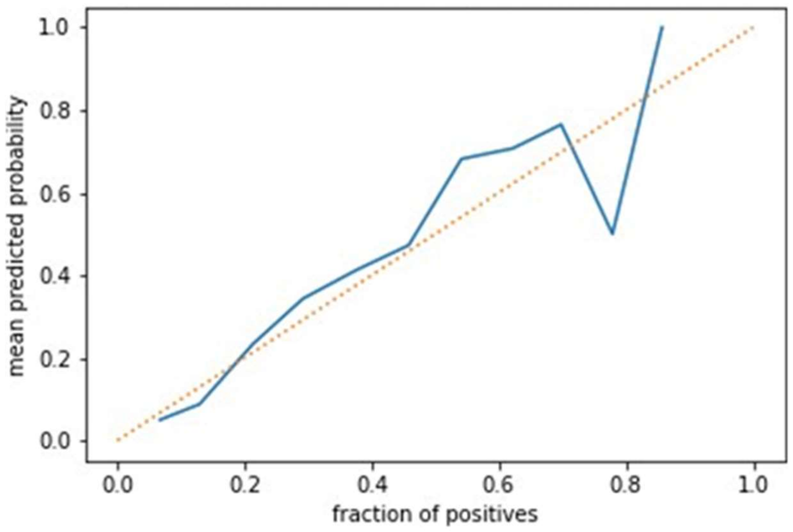

© 2022 Wang R et al. *JAMA Network Open.*

eFigure 6. Calibration Curves for Prediction of No Response by the Logistic Regression and Random Forest Models

Each curve plots the predicted probability of No Response versus the observed proportion of patients with no response in ten strata, ordered by the probability of response. In models with good calibration, the curve should approximate the 45-degree line.

Logistic regression – Full Model

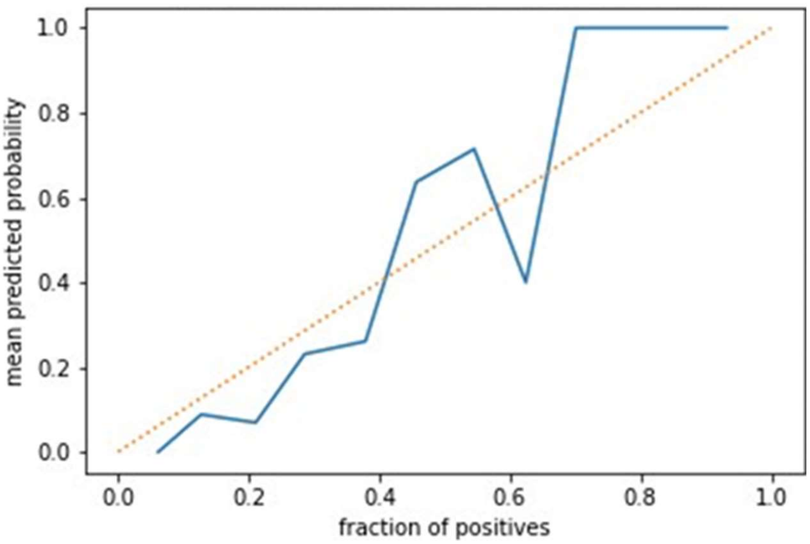

Logistic regression – Reduced Model

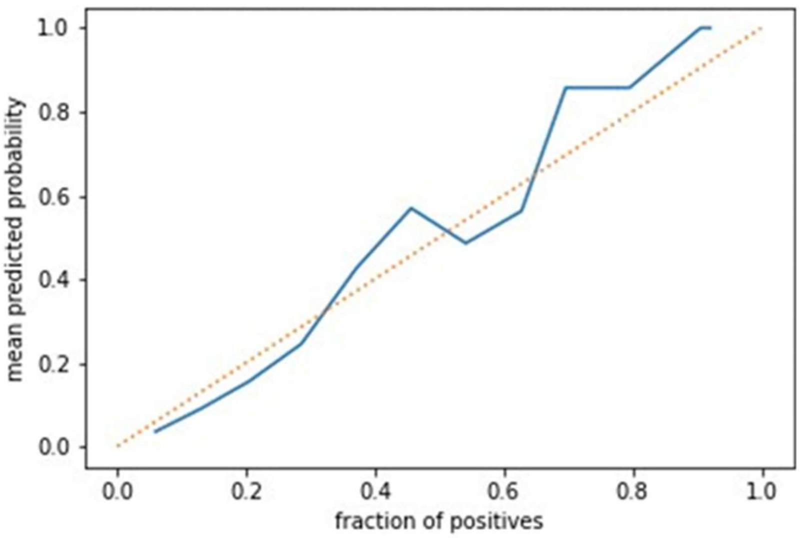

© 2022 Wang R et al. *JAMA Network Open.*

Random Forests – Full Model

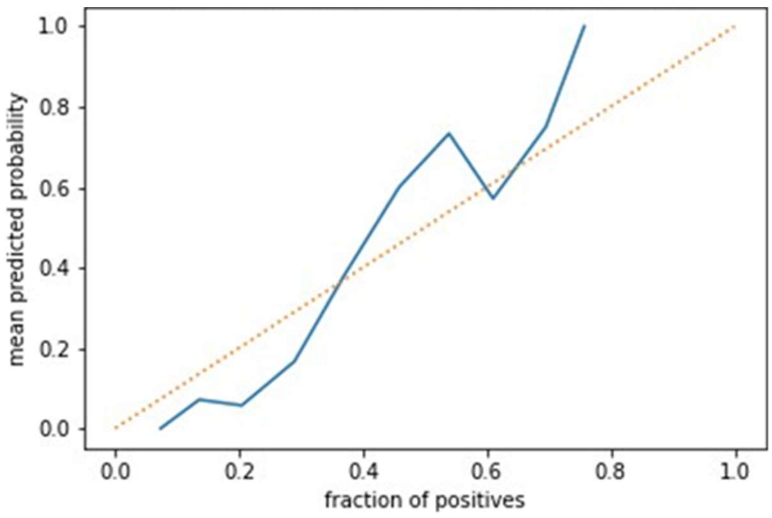

Random Forests – Reduced Model

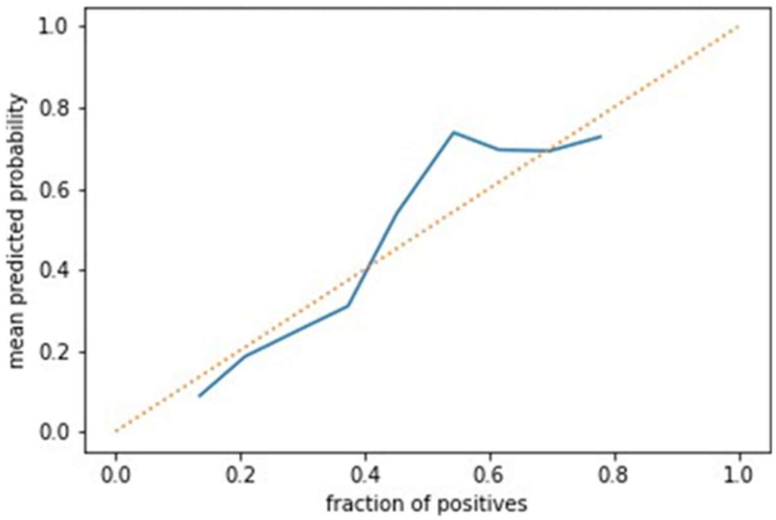

© 2022 Wang R et al. *JAMA Network Open.*

eTable 7. Positive Predictive Values (PPVs) and Negative Predictive Values (NPVS) at Different Prevalences of Major Response and No Response*

	Sensitivity	Specificity	PPV (Prevalence = 0.25)	PPV (Prevalence = 0.5)	NPV (Prevalence = 0.25)	NPV (Prevalence = 0.5)
**Major Response at Week 12**						
LR, Full Model	0.50	0.84	0.51	0.76	0.83	0.63
LR, Reduced Model	0.50	0.86	0.54	0.78	0.84	0.63
RF, Full Model	0.46	0.84	0.49	0.74	0.82	0.61
RF, Reduced Model	0.50	0.89	0.60	0.82	0.84	0.64
**No Response at Week 12**						
LR, Full Model	0.46	0.91	0.63	0.84	0.83	0.63
LR, Reduced Model	0.38	0.92	0.61	0.83	0.82	0.60
RF, Full Model	0.41	0.96	0.77	0.91	0.83	0.62
RF, Reduced Model	0.34	0.95	0.69	0.87	0.81	0.59

*LR: logistic regression; RF: random forest.

© 2022 Wang R et al. *JAMA Network Open.*
